# Metabolomic analysis of a core collection of *Brassica rapa* and *Brassica oleracea* unveils unexpected chemical diversity with potential applications in chemical ecology and breeding

**DOI:** 10.1186/s12870-026-08269-4

**Published:** 2026-02-17

**Authors:** Pauline Le Boulch, Anani Amegan Missinou, Stéphanie Boutet, François Perreau, Maria J. Manzanares-Dauleux, Cyril Falentin, Olivier Filangi, Christine Lariagon, Alain Bouchereau, Mathieu Rousseau-Gueutin, Massimiliano Corso, Antoine Gravot

**Affiliations:** 1 Université de Rennes, Institut Agro, INRAE, IGEPP, Le Rheu, F-35042 France; 2 Université Paris-Saclay, AgroParisTech, INRAE, Institute Jean-Pierre Bourgin for Plant Sciences (IJPB), Versailles, 78000 France; 3 Metabolic Profiling and Metabolomic Platform (P2M2), METABOHUB, BIA-IGEPP, Le Rheu, F- 35000 France

**Keywords:** Brassicaceae, Specialized metabolites, Glucosinolates, MAM, Indole phytoalexins, Blumenol, Flavonol hexosides

## Abstract

**Background:**

Specialized metabolites play key roles in ecological interactions and stress responses, yet their diversification remains poorly understood in many crop species. In the *Brassica* genus, most metabolomic studies have focused on a limited number of compound classes, thereby underestimating the breadth of chemical variation and its evolutionary significance. Here, we use untargeted metabolomics combined with genomic resources to explore how metabolic diversity, enlarged through CuCl_2_ inductive treatments, can be systematically analysed to uncover novel biochemical features and generate testable hypotheses on biochemical innovation across a diverse panel of divergent *Brassica* species, as well as within species.

**Results:**

Using a diverse panel of 10 *B. oleracea* and 10 *B. rapa* accessions, we constructed a curated metabolomic dataset integrating root and shoot metabolomes from plants grown under optimal conditions and exposed to CuCl₂-induced stress responses, followed by manual compound annotation. Available genomic data were used to support mechanistic interpretations of the observed variation among accessions. This approach enabled the exploration of a wide array of *Brassica* metabolites, notably by incorporating underexplored classes such as megastigmanes, phenolamides, and tetra/pentahexosylated acylated flavonoids. We uncovered pronounced inter- and intraspecific metabolic signatures, revealing distinct evolutionary trajectories between the two species. Strikingly, we detected distinct classes of blumenol derivatives across *Brassica* species, a plant genus considered non-mycorrhizal. This finding extends the known occurrence of these compounds and raises new questions about their biological roles. In addition, we link variation in glucosinolate chain-length chemotypes to specific mutations and structural variants in *MAM* genes, illustrating how metabolomic patterns can guide mechanistic genomic investigations.

**Conclusions:**

Together, these results show how a curated metabolomic dataset can simultaneously serve as a reference framework and as a driver for hypothesis-oriented research. By connecting metabolic variation to genomic features, our study provides a basis for functional investigations and offers new opportunities to exploit specialized metabolic traits in breeding programs aimed at improving stress resilience and ecological performance in *Brassica* crops.

**Supplementary Information:**

The online version contains supplementary material available at 10.1186/s12870-026-08269-4.

## Background

The genus *Brassica* encompasses numerous cultivated species, including *Brassica oleracea* and *Brassica rapa* which play prominent roles in human nutrition. The extensive genetic diversity within these two species is reflected in the wide range of cultigroups in *B. rapa* (e.g., turnip, Chinese cabbage, pak choi) and *B. oleracea* (e.g., cauliflower, broccoli, cabbage, Brussels sprouts, kale) [1–3]. This diversity is not only reflected in their morphological traits but also in their phytochemical profiles, which may significantly influence the organoleptic properties and potential health benefits of cultivated varieties.

Thus, research on specialized metabolites in these species has long been a focal point, with significant attention given to glucosinolates (GLSs) and phenolic compounds [4–7]. Among phenolics, flavonoids, particularly derivatives of kaempferol, quercetin and isorhamnetin, are the most abundant and are often characterized by polyhexosylated and polyacylated structures. Polyhexosylated derivatives of hydroxycinnamic acids and quinic acid are also frequently detected [8, 9]. The metabolic profiles of these compounds often vary significantly between the two species or even among cultivars within each species. For instance, hexosylated isorhamnetin is predominantly accumulated in *B. rapa* compared to *B. oleracea* genotypes [10], although further studies expanding the range of accessions will be required to confirm the generality of this pattern.

Glucosinolates are nitrogen-containing compounds classified into four subgroups based on their amino acid precursors: tryptophan-derived indolic GLSs, methionine-derived aliphatic GLSs, phenylalanine/tyrosine-derived phenylalkyl GLSs, and branched-chain amino acid (BCAA)-derived alkyl GLSs [11, 12]. All four subgroups occur in both *B. rapa* and *B. oleracea* [13], yet some GLSs display distinct species-specific distribution patterns. For example, methionine-derived GLSs from *B. oleracea* exclusively display 3–4 C-chain-length, whereas only 4–5 C-chain lengths have been reported in *B. rapa* [14]. In addition, substantial intra-specific variation of the relative abundance of 3C/4C – 4C/5C GLS types has been observed in each species [13]. These variations in aliphatic GLS lateral chain lengths, which have important ecological consequences for herbivory, are largely governed by genetic variation in *Methylthioalkylmalate Synthase* (*MAM*) genes, as extensively documented in *Arabidopsis thaliana* [15, 16] and to a much lower extent in *Brassica* species [17].

Increasing attention has been directed toward the ecological functions of the diverse phytochemicals present in *Brassica* vegetative organs. Flavonoids and GLS compounds have been well studied for their contributions to pathogen and insect resistance [18, 19], insect feeding stimulation [20, 21], recruitment of beneficial root-associated microbes [22], and stimulation of parasitic seed germination [23]. However, beyond GLSs and phenolics, our understanding of specialized metabolism in *Brassica* remains incomplete. Recent advances in metabolomics now provide unprecedented opportunities to expand this knowledge and to investigate agro-ecologically and nutritionally relevant compounds from previously understudied biochemical families.

In this study, our first objective was to characterize both the breadth of specialized metabolite diversity and the dynamics of stress-inducible specialized metabolism in the roots and leaves of a panel of 20 accessions (Supplementary Table S1) representing the main genetic cultivar groups of the crop species *B. oleracea* and *B. rapa*. To this end, we combined untargeted metabolomics with an extensive manual annotation effort, with particular attention to compound families that are usually overlooked or unexpected in this genus, as well as on their inter- and intraspecific distribution patterns. Among these, we focused on several metabolite classes whose occurrence and diversification had not previously been anticipated in *Brassica*, including blumenol derivatives, a subset of megastigmanes (C13-norisoprenoids), which we further explored as a case study of potential biochemical innovation. Finally, because the metabolomic dataset presented in this work constitutes a powerful framework for integrating genetics and metabolomics, we leveraged the available full-length genome sequences of nine genotypes from the *Brassica* diversity panel as an application example to identify potential mutations in specific biosynthetic genes that could explain the distinctive patterns of aliphatic glucosinolates observed in certain accessions.

## Materials and methods

### Plant material and growth conditions

Experiments were conducted on a core collection of 10 *B. rapa* and 10 *B. oleracea* genotypes. Detailed sources of the plant seeds used are given in Supplementary Table S1. All plant accessions used in this study were obtained from certified biological resource centres. Accessions provided by the Center for Genetic Resources (CGN, The Netherlands) and by Chinese institutions were supplied under FAO Standard Material Transfer Agreements (MTA-FAO). French accessions, including the wild *B. oleracea* accession 'Bos01 Le Hode' collected in France, were obtained from the Biological Resource Center BraCySol (France) and are distributed under MTA. The taxonomic identity of all accessions corresponds to the voucher documentation maintained by the respective holding institutes (CGN, BraCySol, and Chungnam National University). Although the original accessions were historically collected from diverse geographical regions, the seed lots used in this study all originated from standardized and controlled seed multiplications conducted in our laboratory. As *B. napus* results from the hybridization and genome doubling between *B. oleracea* and *B. rapa*, we also included one accession of *B. napus* (*var*. Aviso) for comparison. All procedures complied with institutional, national, and international regulations governing plant research and the use of plant genetic resources.

Seeds were sown in a substrate composed of 54% peat, 40% sand, and 6% clay, and grown in a greenhouse under controlled conditions with a 16-hour day/8-hour night photoperiod at 20 °C. Irrigation alternated between a liquoplant nutrient solution and mixed water throughout the 5-week cultivation period. To enhance the metabolic coverage analyzed in this study, half of the plants were subjected to a dual CuCl₂ treatment 48 h before harvest, applied both to the crown (1 mL of 5 mM CuCl₂ per plant) and simultaneously as a foliar spray (5 mM CuCl₂ with Silwett L-77 to facilitate penetration). This method of elicitation is known to mimic pathogen-induced responses [24–26], , and in particular to stimulate the synthesis of various indolic phytoalexins in Brassicaceae [27, 28], providing access to a wide range of inducible metabolites while avoiding potential analytical biases associated with pathogen metabolism or localized reactivity. Moreover, it enables standardized screening across multiple plant accessions, eliminating variability in metabolic responses that could arise from genotype-specific gene-for-gene interactions. After 48 h, the isolated aerial parts of each plant were collected and immediately flash-frozen in liquid nitrogen.

Roots were washed and sampled simultaneously, following the same protocol as for leaves. With the exception of a few samples where germination rates were suboptimal (see Supplementary Table S2), eight plants were consistently pooled per sample to minimize intra-genotypic variability, particularly in cases where the genome is not fixed. For each genotype, three biological replicates were prepared. The harvested samples were subsequently freeze-dried for 100 h and finely ground into a homogeneous powder for downstream metabolite extraction and subsequent UHPLC-HRMS/MS analysis.

### Extraction of polar/semipolar specialized metabolites

Metabolites were extracted from 10 mg of dried powder. Each sample was extracted with 1.8 mL of a solvent mixture consisting of methanol, water, acetone, and trifluoroacetic acid (TFA) (v/v/v/v, 40:32:28:0.05%), containing apigenin as an internal standard (42 ng per mg of dried plant powder). The samples were then subjected to ultrasonic disruption for 20 min at 25 kHz and 4 °C to ensure complete membrane breakdown. These samples were then centrifuged at 20 000 g for 20 min. The process of extraction was performed twice with the same sample and the two resulting supernatants were pooled. 1500 µL were dried for the ESI+ mode and 500 µL were dried for the ESI- mode first in a rotary evaporator and completed in a freeze dryer, resuspended in 200 µL of ULC/MS grade water (Biosolve) with 10% of ULC/MS grade acetonitrile (biosolve) and filtered throught the Whatman type filter. A smaller extract volume was used for ESI- mode to prevent signal saturation from glucosinolates, which ionize very efficiently in negative mode.

QC (Quality Control) samples for both leaves and roots were prepared by pooling 10 µL from each extracted sample for the respective organs. These pooled QC samples underwent the same drying and resuspension steps as the individual samples. For each injection sequence (leaf or root, ESI- or ESI+ mode), QC samples were injected at the beginning and end of the sequence, as well as every 24 h, resulting in 4–5 QC injections per sequence. QC data were used to monitor retention time and mass drifts during the sequence and to filter out features with a coefficient of variation greater than 40%.

### UHPLC-HRMS/MS analysis of polar/semipolar specialized metabolites

Untargeted metabolomic analyses were performed using a UHPLC system (Ultimate 3000, Thermo) coupled to a quadrupole time-of-flight mass spectrometer (Q-Tof Impact II, Bruker Daltonics, Bremen, Germany) equipped with an electrospray ionization (ESI) source operating in both positive and negative modes. Chromatographic separation was achieved on a Nucleoshell RP 18 Plus reversed-phase column (2 × 100 mm, 2.7 μm; Macherey-Nagel) maintained at 40 °C. The mobile phases consisted of (A) 0.1% formic acid in H₂O and (B) 0.1% formic acid in acetonitrile, with a flow rate set at 400 µL/min. The gradient program was as follows: 95% A for 1 min; 95–80% A from 1 to 3 min; 80–75% A from 3 to 8 min; 75–40% A from 8 to 20 min; 0% A held until 24 min; re-equilibration to 95% A from 24 to 27 min, followed by column washing at 30% A and re-equilibration, resulting in a total run time of 35 min. Data-dependent acquisition (DDA) was performed in both ESI modes using the following parameters: capillary voltage, 4.5 kV; nebuliser gas pressure, 2.1 bar; dry gas flow, 6 L/min; and drying gas temperature, 250 °C. Mass spectra were acquired at 8 Hz over an *m/z* range of 70–1300. Stepped fragmentation was applied to enhance MS/MS coverage, with collision RF ranging from 200 to 700 Vpp, transfer time from 20 to 70 µs, and collision energy from 20 to 50 eV. Each acquisition cycle consisted of one full MS scan followed by MS/MS acquisition on the five most intense precursor ions detected in the preceding MS scan. All raw LC–MS data (.mzXML files) generated in this study have been deposited under the dataset identifier MSV000100402 in the MassIVE repository (https://massive.ucsd.edu/ProteoSAFe/static/massive.jsp) and are available with the FTP download link (ftp://massive-ftp.ucsd.edu/v11/MSV000100402/).

### UHPLC-HRMS/MS data processing

Data processing was carried out as described in [29]. The raw .d data files (Bruker Daltonics, Bremen, Germany) were converted to .mzXML format using the MSConvert software from the ProteoWizard package version 3.0 [30]. The converted files were processed using MZmine 2.52 software (http://mzmine.github.io/) for both positive and negative ionization modes.

The chromatograms were built using the ADAP chromatogram builder method [31]. Parameters for this step included a minimum group size of three scans, a group intensity threshold of 1000, a minimum highest intensity of 1500, and a *m/z* tolerance of 10 ppm. Deconvolution was performed using the ADAP wavelets algorithm with a signal-to-noise (S/N) threshold set at 8, a peak duration range between 0.02 and 2 min in ESI+ mode and 0.01 to 2 min in ESI- mode, and a retention time (RT) wavelet range of 0.01 to 0.25 min in ESI+ mode and 0.1 to 0.8 min in ESI- mode. MS/MS scans were paired based on a *m/z* tolerance of 0.01 Da and an RT tolerance of 0.1 min. Peak duration and RT wavelet parameters were empirically optimized based on the observed chromatographic peak widths under the applied UHPLC conditions and validated using QC samples. To group isotopic peaks, the isotopic peak grouper algorithm was applied with a *m/z* tolerance of 10 ppm and an RT tolerance of 0.1 min. Peaks were then filtered using the feature list row filter to retain only those containing MS2 scans. Sample alignment was performed using the join aligner module, with an *m/z* tolerance of 10 ppm, a RT tolerance of 0.1 min, and equal weighting for both *m/z* and RT values.

Curation of the dataset was performed to remove features with a coefficient of variation in QC > 40%, as well as redundant ions corresponding to isotopes or adducts. Adduct curation was carried out using a combined approach: (i) the “Adduct Search” module in MZmine with tolerances of 0.2 min and 0.006 Da, and (ii) a R script described in [32] using the same tolerances. Only adducts identified by both methods were removed. When multiple ion forms were detected for the same compound, only the molecular ions [M + H] + and [M-H]- were retained. During the merging of ESI + and ESI- datasets, redundant ions in ESI- were discarded, retaining only ESI+ ions due to the greater coverage of annotation databases in positive mode.

Compound annotation began with the MZmine identification module using the “custom database search” feature. Matching was performed against a personal library from the IJPB (Jean-Pierre Bourgin Institute for Plant Sciences, Versailles, France), containing 103 annotations in ESI+ mode and 71 in ESI- mode. This step relied solely on *m/z* and retention time, with tolerances of 0.0025 Da (or 6 ppm) and 0.3 min, respectively, as the library did not include MS/MS spectra at the time. These initial matches were subsequently validated through manual inspection of the corresponding MS/MS spectra.

Further annotation was conducted using GNPS molecular networking with standard settings, requiring at least three shared fragment ions and a cosine score of 0.65 or higher. Additional annotations were then obtained by propagation: nodes annotated *via* the internal library or GNPS databases served as starting points for the manual annotation of neighbouring nodes. The reliability of compound identifications was assessed according to the guidelines proposed by [33] and refined by [34], with particular emphasis on fragmentation patterns observed in the MS/MS spectra.

### Statistical analysis

Pre-processed data from the MZmine analysis were subjected to statistical evaluation using MetaboAnalyst and RStudio. Principal Component Analysis (PCA), heatmaps, and volcano plots were generated in MetaboAnalyst [35], applying log transformation and Pareto scaling to normalize the data and enhance interpretability. Significant differentially accumulated between *B. rapa* and *B. oleracea* were identified using a combination of fold-change analysis (FC > 2) and unpaired t-tests, with p-values adjusted for multiple testing using the Benjamini–Hochberg False Discovery Rate (FDR) procedure. Metabolites with an FDR-adjusted p-value < 0.05 were considered statistically significant. Volcano plots and heatmap were generated using metabolites selected based on these inter-species statistical criteria to visualize relative abundance patterns between the two species. In addition, within-species variation across genotypes was assessed separately for each species using one-way ANOVA, with a False Discovery Rate (FDR)-adjusted p-value < 0.05 used as the significance threshold. For further exploration, boxplots were created for all statistically significant and annotated compounds using the R packages “PMCMRplus” and “ggplot2.” The Kruskal-Wallis test was employed to evaluate overall differences among groups, followed by a Conover post-hoc test with Bonferroni adjustment to identify pairwise differences. This robust statistical approach ensured the identification of meaningful metabolic distinctions between the two species while controlling for multiple comparisons.

### Genetic analysis

The protein sequences of MAM1-A, MAM1-B, MAM2-A, and MAM2-B from *Brassica juncea*, as described by [36], were subjected to BLAST analysis against the genomes of various *Brassica oleracea* accessions (‘HDEM’ v1, ‘C10’ v1, ‘CB151’ v2, ‘nd125’ v1, and ‘Bos01 Le Hode’ v1). Sequencing data and associated materials are available in the European Nucleotide Archive (ENA) (PRJEB91446 for ‘C10’, ‘nd125’ and ‘bos01_Le_Hode’ and GCA_900416815.2 for ‘HDEM’), except for ‘CB151’. BLAST analyses were conducted on the Genouest cluster (https://help.genouest.org/usage/cluster) using BLAST+ version 2.9. Protein sequences of *MAM* genes from *Arabidopsis thaliana* were also retrieved for comparison. Genes with at least 30% homology and greater than 80% sequence length similarity to *B. juncea* MAMs were recovered. The selected sequences were aligned using MAFFT in Geneious Prime (2024.1.1, Biomatters Ltd). After visually cleaning the alignement, a maximum parsimony phylogenetic tree was constructed using MEGA11 (version 11.0.13), using a bootstrap method (1,000 replicates). For data processing, the partial deletion method was used with a site coverage cutoff of 90%. The tree was rooted using the *A. thaliana MAM1* gene. A second phylogenetic tree was constructed in the same way as the first, retaining only the sequences closest to the *MAM1* and *MAM2* copies of *Brassica juncea* identified in the first phylogenetic tree.

Geneious Prime was also used to analyze amino acid variations at the catalytic sites of MAM proteins identified in *B. juncea* by [36]. Promoter regions of the identified *B. oleracea MAM* genes were subsequently retrieved and aligned to compare regulatory elements across genotypes.

## Results

### Global patterns of metabolic variation across *Brassica* species, organs, and stress conditions

Metabolomic profiles were realized on *B. rapa* and *B. oleracea* core-collections (Supplementary Table S1), which capture a broad range of genetic and phenotypic diversity within each species. UHPLC-HRMS/MS analyses were performed on both CuCl₂-treated and untreated plants. After automated and manual filtering, we detected 4,863 metabolic features in the leaves (950 ions in ESI- mode ; 3,913 in ESI+ mode) and 3,086 features in the roots (800 ions in ESI- mode ; 2,286 in ESI+ mode) (Supplementary Tables S3 & S4).

**Fig. 1:**
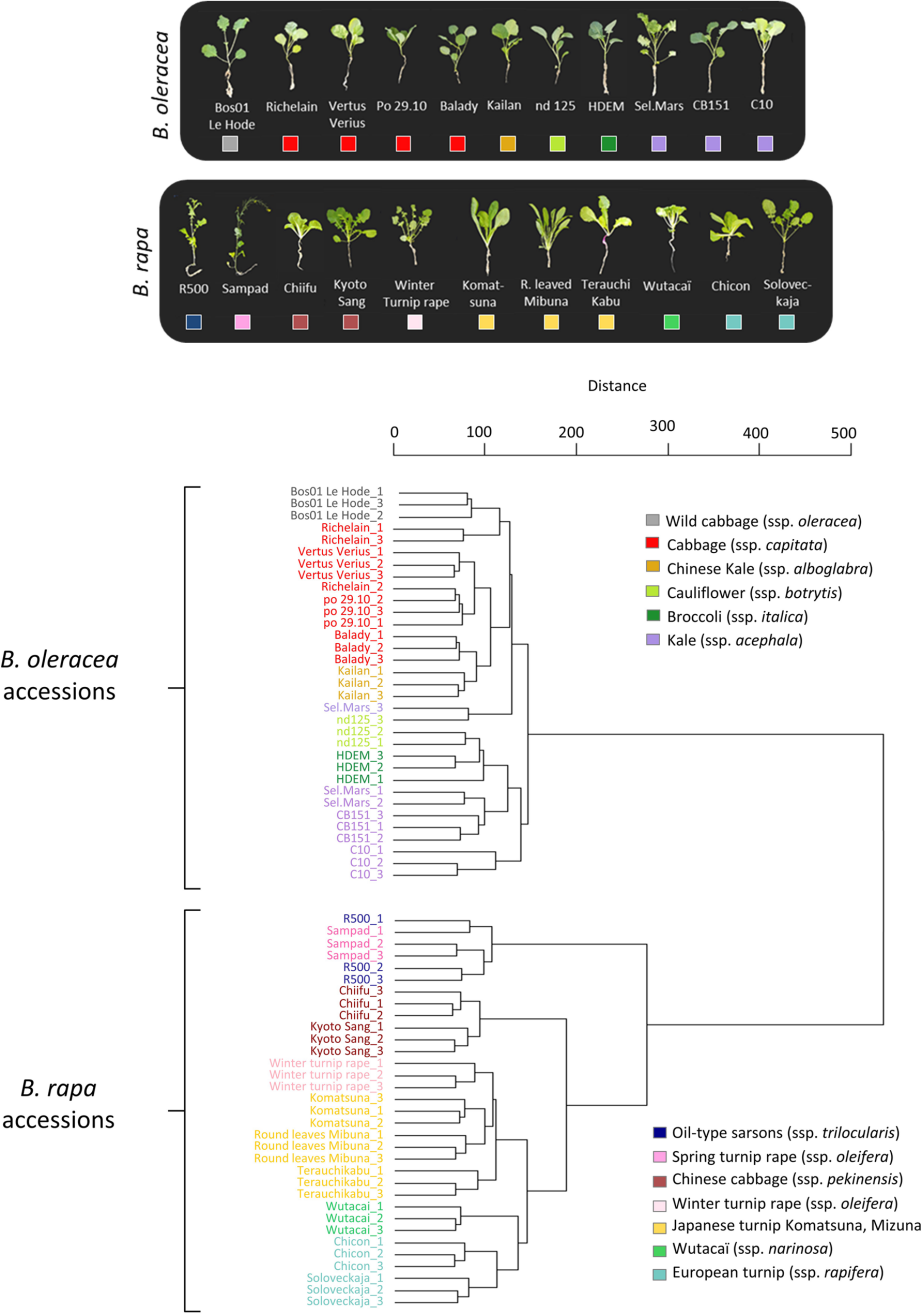
Hierarchical clustering dendrogram of untreated leaf samples based on their metabolomic profiles. Clustering was performed using 4,863 ions detected by UHPLC-HRMS/MS in leaf samples, with data log10-transformed. The Ward’s method and Euclidean distance metric were used to construct the dendrogram. Each color represents the “cultigroup” of the respective accession. Three biological replicates for each accession are displayed in the dendrogram. Images of each genotype at the three-week stage are shown below the dendrogram

**Fig. 2:**
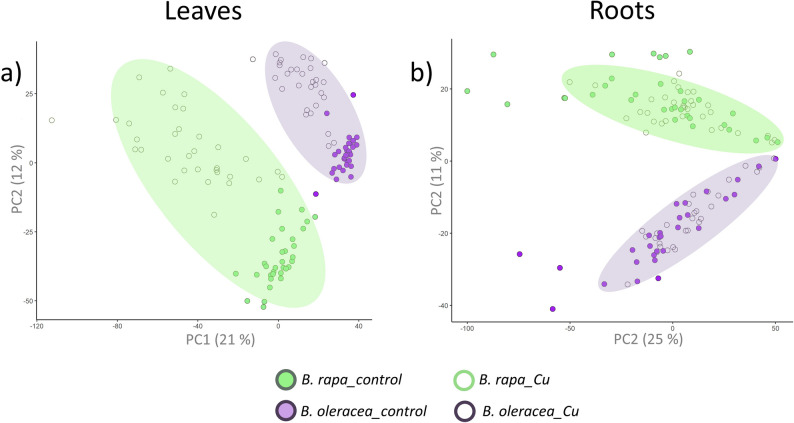
PCA score plot of leaf and root metabolomes in *B. rapa* and *B. oleracea* accessions. PCA score plots constructed from the full untargeted metabolomic dataset obtained by UHPLC-QToF-MS/MS for leaf (**A**) and root (**B**) samples. Green and purple points represent *B. rapa* and *B. oleracea* accessions, respectively. Each point corresponds to one of the three biological replicates per accession

Hierarchical clustering of untreated *B. rapa* or *B. oleracea* accessions based on their leaf metabolomic profiles (Fig. 1) closely matches the phylogenetic classification described by [37]. Accessions were clearly separated by species, and members of the same cultigroup clustered within the same clade. Then, to further explore metabolic variation between species, we performed a Principal Component Analysis (PCA) on all leaf analytical features, providing additional insight into the major chemical sources of variation among the characterized metabolomes (Fig. 2a). These differences were already evident in representative base-peak UHPLC–HRMS chromatograms, which displayed species-specific features as well as CuCl₂-dependent variations (Supplementary Figure A).

Interestingly, a similar metabolomic separation between the two species was also observed in root profiles (Fig. 2b). However, while the first two principal components account for only 36% of the total variance and do not show a clear separation between CuCl₂-treated and untreated roots in two-dimensional projections, statistical analyses indicate that several principal components (PC1, PC4, PC5, PC7, and PC8) are significantly associated with CuCl₂ treatment. Together, these results indicate that CuCl₂ has a subtle effect on the root metabolic profile compared with leaves, and that this effect is distributed across multiple dimensions rather than captured in a single 2D PCA projection.

**Fig. 3:**
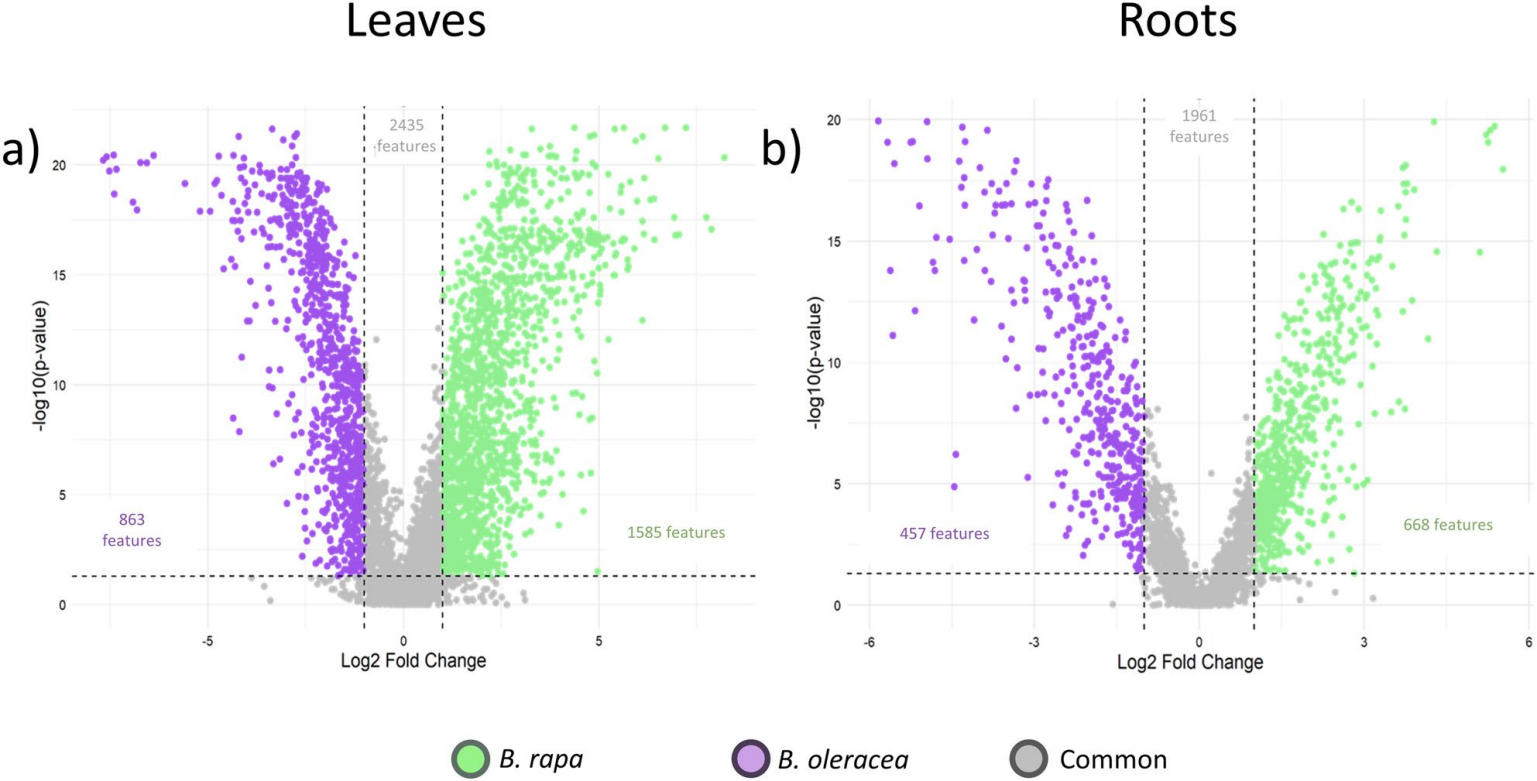
Differential ion accumulation in *B. rapa* and *B. oleracea* leaves and roots revealed by UHPLC-HRMS/MS. Volcano plots showing ion differences in untargeted leaf (**A**) and root (**B**) samples from UHPLC-HRMS/MS analysis. Green and purple circles indicate ions more accumulated *in B. rapa* and *B. oleracea*, respectively. The selection criteria were a fold change greater than 2 and an FDR-adjusted p-value less than 0.05 (Benjamini–Hochberg correction). The x-axis represents the log-transformed fold change, while the y-axis shows the log-transformed adjusted p-value, allowing for the identification of compounds with significant differences in abundance between species

Among the 4,863 features detected in leaves, 2,428 (50%) differed between the two species (Fig. 3a, full list in Supplementary Table S5). Of these, 1,565 features were more abundant in *B. rapa*, including 555 induced by CuCl₂ (37% of the 1,491 CuCl₂-induced features in *B. rapa*). In *B. oleracea*, 863 features accumulated at higher levels, with 136 induced by CuCl₂ (16% of the 1,395 CuCl₂-induced features in *B. oleracea*). In roots, interspecific differences were smaller: 668 features accumulated more strongly in *B. rapa*, and 457 in *B. oleracea* (Fig. 3b, full list in Supplementary Table S6) among which only 28 induced by CuCl₂.

Among the induced compounds, two categories can be distinguished: on the one hand, those already present in non-stressed plants whose abundance increases following CuCl_2_ stimulation, and on the other hand, compounds that are detected only after stimulation. This second category accounts for nearly 15% of the inducible features in leaves (Supplemetary Table S5, and S6, column “CuCl₂-dependent detection”, and see example in Supplementary Figure A.3), demonstrating that CuCl_2_ treatment significantly broadened our knowledge of the metabolic repertoire in this organ. In contrast, this represents only 1% of the inducible features in roots.

### In-depth molecular network–guided metabolite annotation reveals flavonol and Blumenol substitution patterns that clearly discriminate between *B. rapa* and *B. oleracea*

**Fig. 4:**
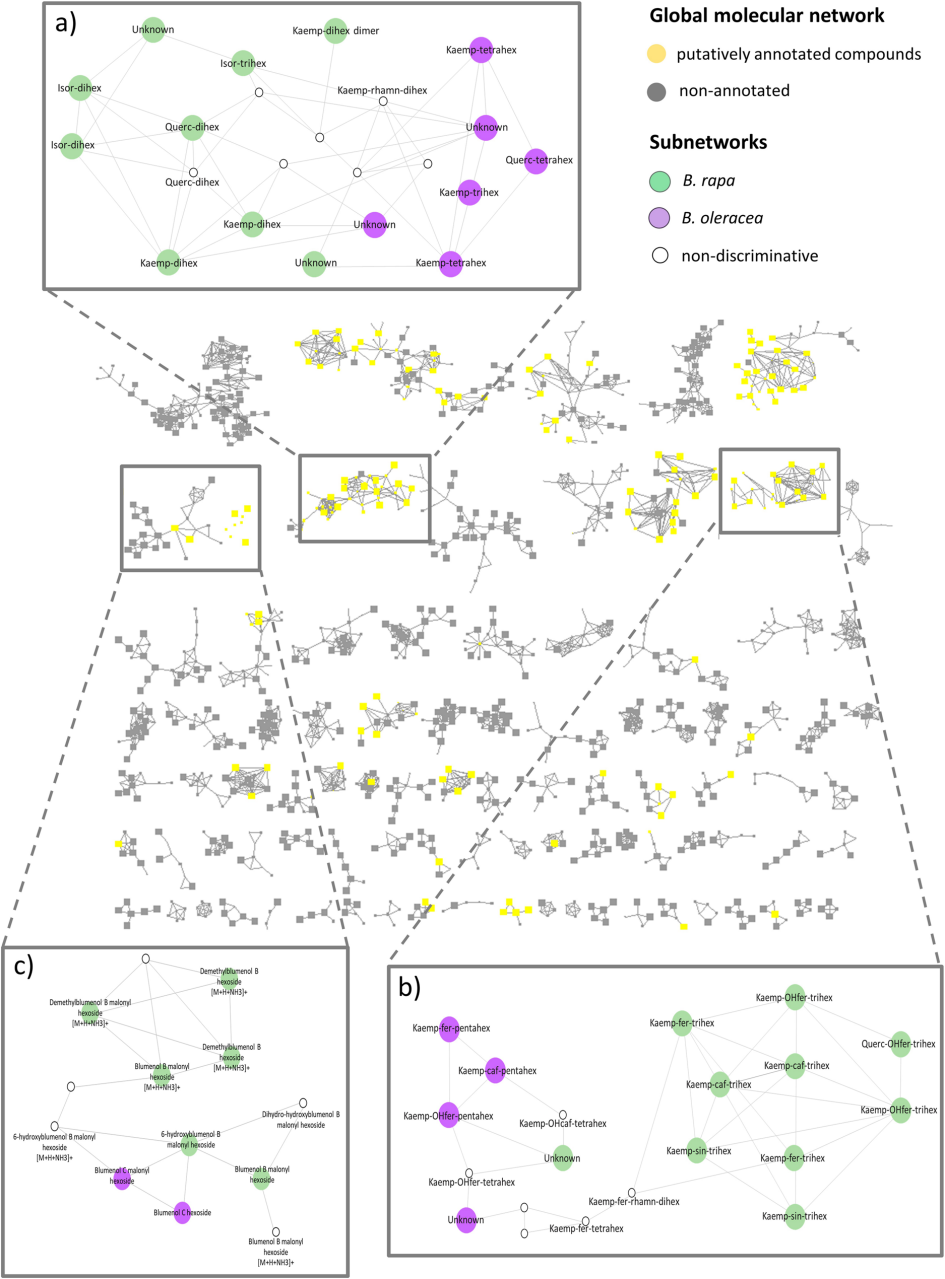
Molecular network of ions detected in ESI+ UHPLC-HRMS/MS from leaf extracts of *B. oleracea* and *B. rapa*. The network was generated using GNPS with a cosine score threshold > 0.65, a minimum of three shared MS/MS fragments, and a maximum of ten edges per feature. In the global network, yellow nodes represent manually annotated ions, while gray nodes indicate non-annotated ions. Subnetworks (**A**) and (**B**) correspond to flavonoid families, and subnetwork (**C**) corresponds to the megastigmane family. Nodes with larger diameters in the subnetworks highlight metabolites exhibiting statistically significant differences in abundance between all *Brassica rapa* accessions (green) and all *Brassica oleracea* accessions (purple) (t-test, *p* < 0.05, FC > 2). Smaller white nodes with black outlines within these subnetworks represent ions shared between both species (non-discriminant). All metabolite annotations are putative (level 2 confidence) based on MS/MS spectral data

Extensive annotation was performed on both discriminant and non-discriminant features. In total, 247 putative compounds were annotated (confidence level 2 according to [33, 34]) across leaves and roots, provigin a more detailed characterization of the phytochemical profiles of the two species. Molecular networking was used to group structurally related compounds into clusters and facilitate annotation (Fig. 4). Each structural annotation was then manually validated through detailed inspection of accurate mass correspondences and MS/MS fragmentation patterns. All annotations, together with ion characteristics and fragmentation data, are provided in Supplementary Table S7. In leaves, 197 putative compounds were annotated, belonging to various chemical families including glucosinolates, hydroxycinnamic acids, flavonoids, coumarins, megastigmanes (C13-norisoprenoids), amino acids and derivatives, fatty acids, phenolamides, oxylipins and indole derivatives. Among them, 138 discriminated between the two *Brassica* species (Supplementary Table S5). In roots, 50 compounds were putatively annotated, of which 23 enabled species discrimination (Supplementary Table S6).

Phenolic compounds, particularly glycosylated flavonoids, are well-represented among the annotated discriminant metabolic features in leaves and have proven to be reliable markers of metabolic diversity between *Brassica oleracea* and *Brassica rapa*. As shown in Fig. 4a, b and Supplementary Table S5, among the seven annotated glycosylated flavonoids found at higher levels in *B. oleracea*, six were hexosylated kaempferol derivatives and one was derived from quercetin. In contrast, *B. rapa* accumulated substantially higher levels of 22 hexosylated kaempferol derivatives (distinct from the six previously mentioned), three glycosylated quercetin derivatives, and four hexosylated isorhamnetin derivatives. Notably, compounds p1327 (isorhamnetin hexoside) and p1305 (isorhamnetin dihexoside) were more than 100-fold more abundant in *B. rapa* than in *B. oleracea* (Supplementary Table S5), making them the most discriminant markers between the two species. Additionally, the degree of glycosylation emerges as a key discriminant factor. While *B. oleracea* accessions are distinguished by a characteristic pattern of pentahexosylated flavonols, *B. rapa* accessions preferentially accumulate di- and trihexosylated flavonols (Fig. 4b, Supplementary Table S5).

As highlighted in Fig. 4c, our analyses also identified two megastigmane derivatives, blumenol C malonyl hexoside and blumenol C hexoside, which accumulated at high levels exclusively in the leaves of *B. oleracea* (Fig. 4c, Supplementary Table S5). This annotation was initially prompted and supported by the prior identification of these compounds in maize samples in a previous study conducted in our laboratory using the same analytical workflow (see details in the M&M section). Because, to our knowledge, blumenol derivatives have rarely been reported in Brassicaceae, this unexpected observation motivated a more detailed exploration of the corresponding metabolic subnetworks (Fig. 4c). To annotate these metabolomic features, we conducted an in-depth analysis of their MS/MS fragmentation patterns, combining comparative spectral analysis with an interpretation of the underlying fragmentation schemes (Supplementary Figure B and Table 1 “MS2 interpretation”).

**Table 1:** Structural annotation of a subset of metabolite markers associated with intraspecific diversity in *Brassica Rapa* and *Brassica Oleracea* accessions

ID	putative name	formula	chemical family (ClassyFire)	Identification level (MSI)	ionisation	adduct type	Organes	MS2 interpretation
n902	Biscoumaroyl spermidine	C25H31N3O4	Phenolamines	2	negative	[M-H]-	Roots	**119.050**(C8H7O)=[M-H-317.173(C17H23N3O3)]-
p1869	Blumenol C-glucosyl-malonic acid	C22H34O10	Megastigmanes	2	positive	[M + H]+	Leaves	**211.169**(C13H23O2)=[M + H-162.053(C6H10O5)-86.000(C3H2O3)]+ ; **193.159**(C13H21O)=[M + H-162.053(C6H10O5)-86.000(C3H2O3)-18.01(H2O)]+ ; **137.096**(C9H13O)=[M + H-162.053(C6H10O5)-86.000(C3H2O3)-18.01(H2O)-56.062(C4H8)]+
p4740	Blumenol B malonyl glucoside	C22H34O11	Megastigmanes	2	positive	[M + H]+	Leaves	**227.165**(C13H23O3)=[M + H-162.053(C6H10O5)-86.000(C3H2O3)]+ ; **209.153**(C13H21O2)=[M + H-162.053(C6H10O5)-86.000(C3H2O3)-18.01(H2O)]+ ; **191.143**(C13H19O)=[M + H-162.053(C6H10O5)-86.000(C3H2O3)-2xH2O(18.01)]+ ; **173.132**(C13H17)=[M + H-162.053(C6H10O5)-86.000(C3H2O3)-3xH2O(18.01)]+
p2448	Demethylblumenol B malonyl glucoside	C21H32O11	Megastigmanes	2	positive	[M + H+NH3]+	Leaves	**195.138**(C12H19O2)=[M + H-162.053(C6H10O5)-86.000(C3H2O3)-18.01(H2O)-17.026(NH3)]+ ; **213.148**(C12H21O3)=[M + H-162.053(C6H10O5)-86.000(C3H2O3)-17.026(NH3)]+ ; **135.116**(C10H15)=[M + H-162.053(C6H10O5)-86.000(C3H2O2)-18.01(H2O)-60.021(C2H4O2)-17.026(NH3)]+
n555	Polynitrogen heterocycle conjugated to homoleucine	C14H17N5O5	-	2	negative	[M-H]-	Roots	**290.124**(C13H16N5O3)=[M-H-43.989(CO2)]- ; **206.032**(C7H4N5O3)=[M-H-128.084(C7H12O2)]- ; **163.026**(C6H3N4O2)=[M-H-171.089(C8H13NO3)]-
p1266	Polynitrogen heterocycle conjugated to homophenylalanine	C17H15N5O5	-	2	positive	[M + H]+	Roots	**163.026**(C6H3N4O2)=[M + H-207.089(C11H13NO3)]+ ; **324.109**(C16H14N5O3)=[M + H-46.005(CH2O2)]+ ; **191.02**(C7H3N4O3)=[M + H-179.094(C10H13NO2)]+
n27	Polynitrogen heterocycle conjugated to leucine	C13H15N5O5	-	2	negative	[M-H]-	Roots	**163.026**(C6H3N4O2)=[M-H-157.074(C7H11NO3)]- ; **276.111**(C12H14N5O3)=[M-H-43.989(CO2)]- ; **206.031**(C7H4N5O3)=[M-H-114.068(C6H10O2)]
p1390	Polynitrogen heterocycle conjugated to phenylalanine	C16H13N5O5	-	2	positive	[M + H]+	Roots	**310.094**(C15H12N5O3)=[M + H-46.005(CH2O2)]+; **163.026**(C6H3N4O2)=[M + H-193.074(C10H11NO3)]+ ; **191.021**(C7H3N4O3)=[M + H-165.078(C9H11NO2)]+
p1668	Caffeoyl-malate	C13H12O8	Hydroxycinnamic acids and derivatives	2	positive	[M + H]+	Leaves	**163.038**(C9H7O3)=[M + H-134.021(C4H6O5)]+ ; **135.045**(C8H7O2)=[M + H-162.016(C5H6O6)]+
n218	Coumaroyl-malate	C13H12O7	Hydroxycinnamic acids and derivatives	2	negative	[M-H]-	Leaves	**163.040**(C9H7O3)= [M-H-116.011(C4H4O4)]-
n213	Feruloyl-malate	C14H14O8	Hydroxycinnamic acids and derivatives	2	negative	[M-H]-	Leaves	**193.050**(C10H9O4)=[M-H-116.011(C4H4O4)]- ; **133.015**(C4H5O5)=[M-H-176.047(C10H8O3)]-
p2664	Sinapoyl-malate_isomer	C15H16O9	Hydroxycinnamic acids and derivatives	2	positive	[M + H]+	Leaves	**207.065**(C11H11O4)=[M + H-134.021(C4H6O5)]+ ; **177.055**(C10H9O3)=[M + H-164.032(C5H8O6)]+
p1019	Kaempferol-feruloyl-rhamnoside-dihexoside	C43H48O23	Flavonoid glycoside	2	positive	[M + H]+	Leaves	**177.055**(C10H9O3)=[M + H-2 × 162.053(C6H10O5)-146.058(C6H10O4)-286.047(C15H10O6)]+ ; **287.055**(C15H11O6)=[M + H-2 × 162.053(C6H10O5)-146.058(C6H10O4)-176.047(C10H8O3)]+ ; **339.107**(C16H19O8)=[M + H-162.053(C6H10O5)-146.058(C6H10O4)-286.047(C15H10O6)]+ ; **433.113**(C21H21O10)=[M + H-2 × 162.053(C6H10O5)-176.047(C10H8O3)]+
p1042	Kaempferol-rhamnoside-dihexoside	C33H40O20	Flavonoid glycoside	2	positive	[M + H]+	Leaves	**595.165**(C27H31O15)=[M + H-162.053(C6H10O5)]+ ; **449.107**(C21H21O11)=[M + H-162.053(C6H10O5)-146.058(C6H10O4)]+ ; **433.112**(C21H21O10)[M + H-2 × 162.053(C6H10O5)]+ ; **287.055**(C15H16O6)[M + H-2 × 162.053(C6H10O5)-146.058(C6H10O4)]+
p1041	Indolacetamide	C10H10N2O	Indoles and derivatives	2	positive	[M + H]+	Leaves	**130.065**(C9H8N)=[M + H-45.021(CH3NO)]+ ; **103.055**(C8H7)=[M + H-72.031(C2H4N2O)]+
p3451	Indole acetaldoxime	C10H10N2O	Indoles and derivatives	2	positive	[M + H]+	Leaves	**130.065**(C9H8N)=[M + H-45.021(CH3NO)]+ ; **160.062**(C9H8N2O)=[M + H-15.024(CH3)]+
p2963	Indole acetic acid	C10H9NO2	Indoles and derivatives	2	positive	[M + H]+	Leaves	**130.065**(C9H8N)=[M + H-46.005(CH2O2)]+ ; **158.059**(C10H8NO)=[M + H-18.01(H2O)]+ ; **161.046**(C9H7NO2)=[M + H-15.024(CH3)]+
p2009	Indole carboxylic acid	C9H7NO2	Indoles and derivatives	2	positive	[M + H]+	Leaves	**116.049**(C8H6N) = M + H-46.005(CH2O2)]+ ; **144.043**(C9H6NO)=[M + H-18.01(H2O)]+
p380	Indole carbaldehyde	C9H7NO	Indoles and derivatives	2	positive	[M + H]+	Leaves	**118.065**(C8H8N)=[M + H-27.995(CO)]+
p57	Indolylmethylthiohydroximate	C10H10N2OS	Indoles and derivatives	2	positive	[M + H]+	Leaves	**130.065**(C9H8N)=[M + H-76.993(CH3NOS)]+ ; **117.057**(C8H7N)=[M + H-90.001(C2H4NOS)]+ ; **174.0365**(C10H8NS)=[M + H-33.021(NH3O)]+
n510	Indole carboxylic acid-hexose	C15H17NO7	Indoles and derivatives	2	negative	[M-H]-	Leaves	**160.040**(C9H6NO2)=[M-H-162.053(C6H10O5)]- ; **116.051**(C8H6N)=[M-H-162.053(C6H10O5)-43.99(CO2)]-
p2887	1-aci-nitro-2-(1 H-indol-3-yl)ethane	C10H10N2O2	Indoles and derivatives	2	positive	[M + H]+	Leaves	**116.049**(C8H6N)=[M + H-75.032(C2H5NO2)]+ ; **130.065**(C9H8N)=[M + H]-61.016(CH3NO2)]+
p95	Desulfoglucobrassicin	C16H20N2O6S	Glucosinolates	2	positive	[M + H]+	Leaves	**207.059**(C10H11N2OS)=[M + H-162.053(C6H10O5)]+ ; **130.065**(C9H8N)=[M + H-162.053(C6H10O5)-76.993(CH3NOS)]+
n54	Glucobrassicin	C16H20N2O9S2	Glucosinolates	2	negative	[M-H]-	Leaves	**274.990**(C6H11O8S2)=[M-H-172.066(C10H8O1N2)]- ; **195.033**(C6H11O5S)=[M-H-252.021(C10H8N2O4S)]- ; **96.959**(HSO4)=[M-H-350.095(C16H18N2O5S)]-
n288	4-Hydroxyglucobrassicin	C16H20N2O10S2	Glucosinolates	2	negative	[M-H]-	Leaves	**96.959**(HSO4)=[M-H-366.089(C16H18N2O6S)]- ; **195.032**(C6H11O5S)=[M-H-268.015(C10H8N2O5S)]-
n628	1-Hydroxyglucobrassicin	C16H20N2O10S2	Glucosinolates	2	negative	[M-H]-	Leaves	**96.959**(HSO4)=[M-H -366.088(C16H18N2O6S)]-
p2695	Methoxyindolecarbinole	C10H11NO2	Indoles and derivatives	2	positive	[M + H]+	Leaves	**146.0603**(C9H8NO)=[M + H-32.026(CH4O)]+
p2328	Spirobrassinin_isomer	C11H10N2OS2	Indoles and derivatives	2	positive	[M + H]+	Leaves	**203.027**(C10H7N2OS)=[M + H-48.003(CH4S)]+ ; **178.032**(C9H8NOS)=[M + H-72.998(C2H3NS)]+
p3084	Raphanusamic acid	C4H5NO2S2	Amino acids, peptides, and analogues	2	positive	[M + H]+	Leaves	**117.977**(C3H4NS2)=[M + H-46.005(CH2O2)]+

A database listing all the metabolites putatively identified, their structural annotation, and the source of annotation (internal library, GNPS, or manual curation) is available in Supplementary Table S7. Additional details, including the full dataset with chemical identifiers can be found in the accompanying data files

Blumenol C malonyl hexoside [M + H] + *m/z* 459.221 (C22H35O10+) was characterized by a diagnostic fragment at *m/z* 211.169, corresponding to the protonated aglycone C13H23O2 + after loss of a malonylhexoside moiety, followed by a secondary fragment C13H21O + at *m/z* 193.159 resulting from water loss and double-bond formation on the side chain. In contrast, blumenol B malonyl hexoside [M + H] + *m/z* 475.218 (C22H35O11+) showed a distinct fragmentation behavior, with a major ion C13H21O2 + at *m/z* 209.153 attributed to the loss of a malonylhexoside followed by a cyclization involving the hydroxyl group at C6. Such a propensity for intramolecular cyclization of blumenol B has been previously documented in chemical studies addressing its reactivity and transformation pathways [38]. Subsequent fragmentation led to an ion C13H19O + at *m/z* 191.143, consistent with an additional dehydration step and further unsaturation of the side chain. These differences provide clear MS/MS markers distinguishing blumenols B and C. A compound tentatively annotated as 6-hydroxyblumenol B malonyl hexoside displayed a fragmentation pattern closely related to that of blumenol B, with a systematic + 2 Da shift of the diagnostic fragments, consistent with the replacement of a carbonyl group by a hydroxyl group. Although its retention time was close to that of blumenol C malonyl hexoside, its accumulation profile across samples mirrored that of blumenol B derivatives and differed markedly from blumenol C, arguing against its assignment as a simple [M + H₂O + H]⁺ adduct. Finally, the dihydro-hydroxyblumenol B malonyl hexoside [M + H] + *m/z* 477.234 (C22H37O11+) was annotated based on the presence of characteristic fragments at *m/z* 213.184 (C13H25O2+) and 195.174 (C13H23O+), showing a + 4 Da shift relative to blumenol B, consistent with both hydroxylation and saturation of the endocyclic double bond. Together, these diagnostic fragmentation patterns support the proposed annotation of multiple, structurally distinct blumenol derivatives in *B. rapa* and *B. oleracea*.

Ultimately, these species-specific accumulation patterns reveal a clear partitioning of blumenol metabolism, with blumenol C–derived compounds associated with *B. oleracea*, whereas blumenol B and A derivatives predominate in *B. rapa* (Fig. 4c).

### Hierarchical clustering of annotated metabolites reveals multi-class interspecific metabolic differentiation

**Fig. 5:**
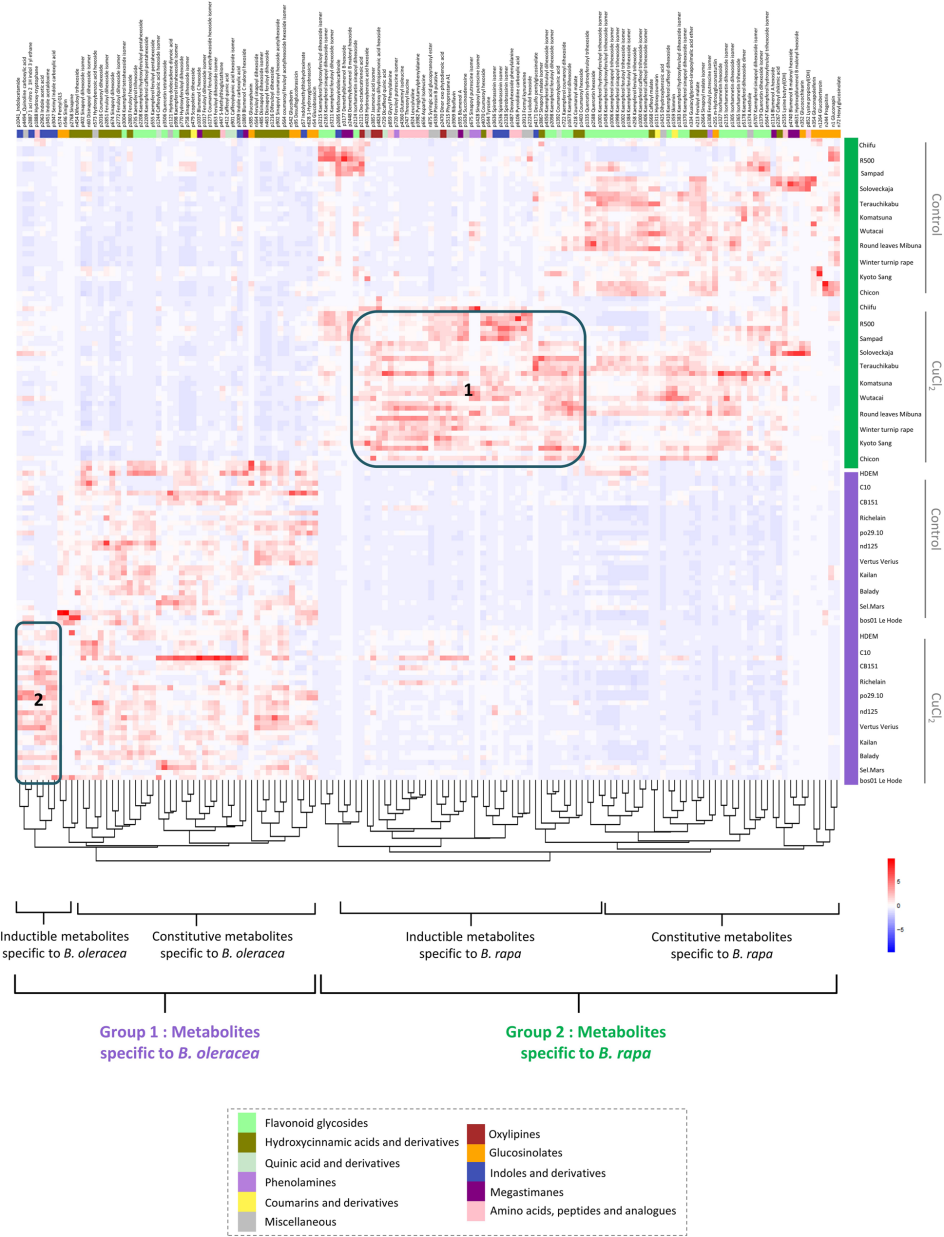
Hierarchical clustering of leaf metabolites with significant interspecific differences in *B. rapa* and *B. oleracea*. Hierarchical clustering analysis (HCA) of leaf metabolites showing significant differences between *B. rapa* and *B. oleracea* species (t-test, *p* < 0.05; fold change > 2). HCA was performed on log-transformed data normalized using Pareto scaling (mean-centered and divided by the square root of the standard deviation), applying Euclidean distance and the Ward algorithm. Each genotype is represented by three biological replicates under control and CuCl₂-treated conditions, except for “PBD20-Bos01 Le Hode”, which includes only one treated sample. Colored squares indicate metabolite chemical families assigned by ClassyFire. Numbered zones (1–4) highlight metabolite clusters discussed in the main text. All metabolite annotations are putative (level 2 confidence) based on MS/MS spectral data

To further investigate these interspecific differences, hierarchical clustering was performed on the 138 annotated discriminant leaf compounds between the two species (Fig. 5). The prominence of putative phenolic compounds is striking, which can be partly explained by their strong representation in databases and their well-clustered patterns in molecular networks, thereby facilitating annotation. Significant differences were observed not only for flavonols but also for hydroxycinnamic acids (HCAs). Among the 16 HCAs specific to *B. oleracea*, most of them are conjugated with one or two hexoses. In contrast, of the 11 HCAs identified in *B. rapa*, six are conjugated with malate, three with putrescine (a polyamine), and only two with hexoses.

The CuCl₂ treatment elicited the accumulation of 41 inducible compounds that were more abundantly produced in *B. rapa* compared to *B. oleracea* (Fig. 5, Box 1). Noteworthy among these were nine putative dipeptides, most of which included either phenylalanine or leucine/isoleucine paired with other amino acids, three phenolamines (with putrescine residues), several oxylipin-related compounds (e.g., jasmonic acid, hydroxy-dihydrojasmonic acid hexoside, and dinor-oxo-phytodienoic acid). This list of inducible compounds also included three isomers of spirobrassinin, which is an indolic phytoalexin that functions primarily as an inducible chemical defence molecule in cruciferous plants [28]. In *B. oleracea*, although fewer inducible annotated metabolites were identified (Fig. 5, Box 2), indolic compounds constituted the predominant class.

Finally, similar comparative analyses were conducted on the metabolomic datasets obtained from root samples (Supplementary Figure C, Supplementary Table S6). This approach revealed a lower number of annotated species-specific compounds compared to the leaf metabolic dataset. However, a cluster of metabolomic features showing similar structure captured our attention. These compounds might correspond to polynitrogen heterocycles conjugated to amino acids [39]. In addition, they were found almost exclusively in roots of *B. rapa* accessions, and were scarcely detectable in *B. oleracea* accessions. The heterogeneity of their distribution among *B. rapa* accessions is discussed in the next section.

### Beyond interspecific differences, chemical variability is expressed at the intraspecific and cultigroup levels

The *Brassica* core collection used in this study encompassed a wide range of cultigroups. This extensive genetic diversity within each of the two species, was expected to be reflected in substantial intraspecific metabolic variation, at least at the quantitative level. In *B. rapa*, 2,588 (53%) and 633 (20%) features showed differential accumulation between at least two accessions of the panel in leaves and roots, respectively. *B. oleracea* accessions appeared slightly less contrasted, with 1,323 (27%) and 482 (16%) features exhibiting differential accumulation between at least two accessions in leaves and roots, respectively.

**Fig. 6:**
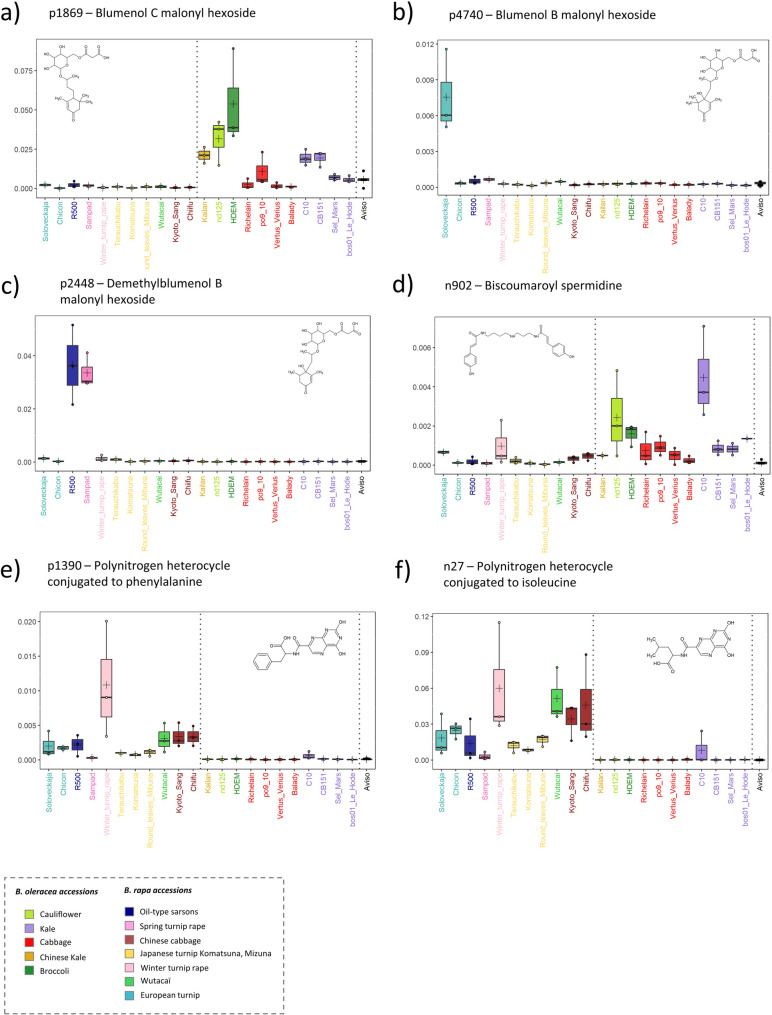
Distribution patterns of six rarely described, putatively annotated metabolites in *Brassica* species. Each boxplot illustrates the distribution of a specific metabolite across panels of *B. rapa*, *B. oleracea*, and *B. napus* accessions. Dashed lines within the boxplots separate accessions by species. Colors indicate the “cultigroup” of each accession. Metabolites p1390, n27, and n902 are root-specific, whereas p1869, p4740, and p2448 are leaf-specific. Hypothetical 2D structures of each metabolite are shown for reference. All metabolite annotations are putative (level 2 confidence) based on MS/MS spectral data

Compounds belonging to the megastigmane family, which are asymmetrically distributed between *B. oleracea* and *B. rapa* as mentioned above, were among those displaying the most pronounced intraspecific variations in accumulation patterns. In *B. oleracea*, blumenol C malonyl hexoside accumulated at high levels in the leaves of ‘nd125’ and ‘HDEM’, both of which belong to closely related phylogenetic cultigroups (cauliflower and broccoli), whereas its abundance was markedly lower in the four accessions of the cabbage cultigroup (Fig. 6a). However, this compound showed contrasting levels among the three kale accessions, being abundant in ‘C10’ and ‘CB151’ but much less in ‘Sel Mars’, suggesting that cultigroup affiliation is not the primary determinant of its accumulation. In *B. rapa*, blumenol B malonyl hexoside (Fig. 6b) was detected at high levels exclusively in the ‘Soloveckaja’ accession, whereas demethylated blumenol B derivatives were predominantly observed in the accessions ‘R500’ and ‘Sampad’, which belong to distinct cultigroups (Fig. 6c).

Similarly, the distribution of phenolamine biscoumaroyl spermidine concentrations in roots (Fig. 6d) was highly contrasted across both the *B. oleracea* and *B. rapa* sub-panels. For example, this compound was found to be four times more abundant in the accession ‘C10’ than in the other two kale accessions. Also, a set of four root metabolomic features annotated as polynitrogen heterocycle derivatives conjugated to phenylalanine, homophenylalanine, leucine, and homoleucine (Fig. 6e, f; Table 1 and Supplementary Table S7) was exclusively detected in root samples from *B. rapa* accessions. However, their accumulation patterns varied substantially among accessions. For example, the phenylalanine conjugate (n1390) was highly abundant in the ‘Winter Turnip Rape’ accession but was present at much lower levels in ‘Sampad’ and in the three Japanese turnip accessions (‘Terauchikabu,’ ‘Komatsuna,’ and ‘Round Leaves Mibuna’).

**Fig. 7:**
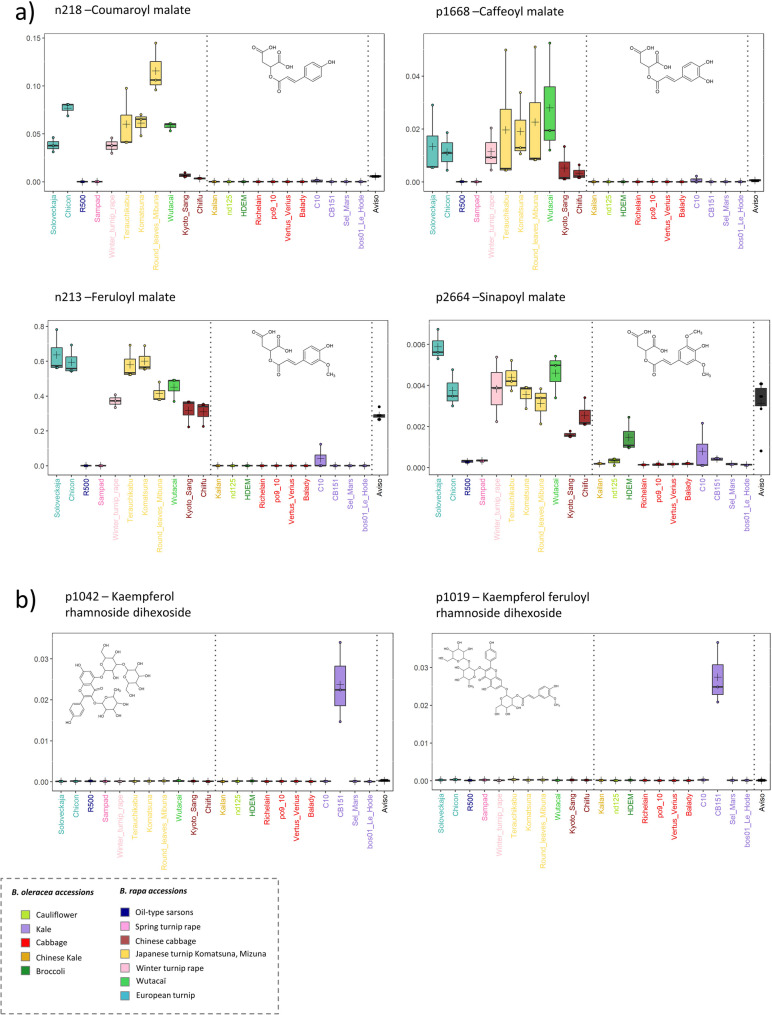
Distribution of leaf metabolites across *Brassica* accessions highlighting accession-specific patterns. Boxplots illustrate the distribution of leaf metabolites across *B. rapa*, *B. oleracea*, and *B. napus* accessions. Each boxplot represents a specific metabolite: (**a**) metabolites scarce or absent in the ‘R500’ and ‘Sampad’ accessions, and (**b**) metabolites unique to a single accession. Dashed lines separate accessions by species, and colors indicate the “cultigroup” of each accession. Hypothetical 2D structures of each metabolite are shown for reference. All metabolite annotations are putative (level 2 confidence) based on MS/MS spectral data

The absence of HCA-malate conjugates, which is the most striking similar metabolic pattern shared by ‘R500’ and ‘Sampad’ (also visible in Fig. 5), is detailed in the four boxplots in Fig. 7a. This reveals that, for these metabolites, the genotypes ‘R500’ and ‘Sampad’ (both *B. rapa*) exhibit profiles more similar to those of *B. oleracea* accessions. Interestingly, the *B. napus* genotype ‘Aviso’ displayed an intermediate pattern, characterized by a cabbage-like accumulation of feruloyl malate and sinapoyl malate, alongside a turnip-like scarcity of coumaroyl malate and caffeoyl malate.

Finally, two flavonol derivatives, kaempferol rhamnoside dihexoside (p1042) and kaempferol feruloyl-rhamnoside dihexoside (p1019) (see Table 1 and Supplementary Figure D for structural annotation), are presented as representative examples of putatively annotated compounds that specifically accumulated in a single accession, namely in the leaves of *B*. *oleracea* ‘CB151’ (Fig. 7b). The proposed rhamnosylation of these kaempferol derivatives is supported by high-resolution MS/MS fragmentation analysis. Both compounds exhibit a characteristic neutral loss of 146.057 Da, corresponding to a deoxyhexose moiety. While this loss could in principle correspond to rhamnose or fucose, rhamnose is considered the most likely assignment based on its prevalence in flavonoids, whereas fucose has been reported only rarely in specialized metabolites, e.g., triterpenoid saponins [40]. Both compounds also display shared diagnostic product ions, including the fragment at *m*/*z* 433.114, indicative of a kaempferol–rhamnoside substructure (Supplementary Figure D, Table 1). The recurrence of these deoxyhexose-specific fragmentation features across structurally related compounds further supports the annotation of rhamnosylated kaempferol derivatives. In the absence of reference standards, these structural assignments should be considered putative (confidence level 2 according to [33, 34]) and based on MS/MS evidence.

### Copper treatment highlights species- and accession-specific patterns of Indole metabolism among *Brassica* accessions

**Fig. 8:**
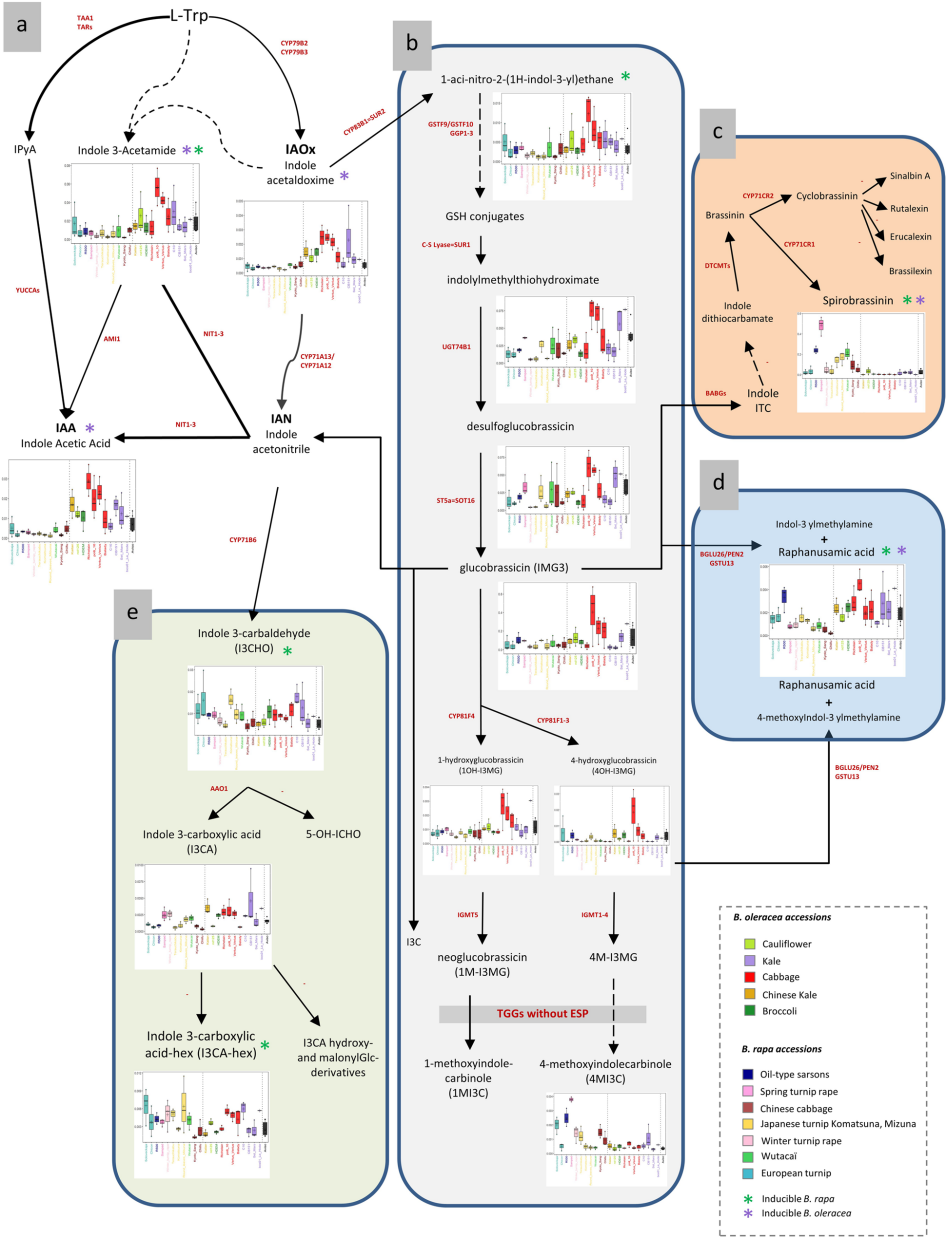
Intraspecific variation of inducible tryptophan-derived indolic metabolites in *B. rapa* and *B. oleracea* leaves. This schematic representation was constructed based on knowledge gathered from *Arabidopsis* and *Brassica* models for (**a**) the auxin biosynthetic pathway [43, 44], (**b**) the indolic glucosinolate pathway [44], (**c**) *Brassica* phytoalexin biosynthesis, (**d**) raphanusamic acid biosynthesis [45, 46], and (**e**) the indole carboxylic acid and derivatives pathway [42]. Most of the genes shown have been characterized in *Arabidopsis*, except for those in pathway (c), which have been identified in *Brassica rapa* [28, 47]. Asterisks (*) indicate compounds induced by CuCl₂ in *B. rapa* (green) and/or *B. oleracea* (purple). Boxplots represent compound concentrations after CuCl₂ treatment, while boxplots for untreated samples are provided in Supplementary Figure E for comparison. All metabolite annotations are putative (level 2 confidence) based on MS/MS spectral data

A feature putatively corresponding to the plant hormone indole-3-acetic acid (IAA), along with features corresponding to two of its known precursors, indole-3-acetamide and indole acetaldoxime, were identified in the metabolomic dataset (Fig. 8a). This latter compound is also a central precursor for the biosynthesis of several indole-derived specialized metabolites found in *B. rapa* and *B. oleracea*. These included three indolic GLS (glucobrassicin, 1- and 4-hydroxyglucobrassicin), three of the biosynthetic intermediates involved in the indole-GLS biosynthesis (i.e., 1-aci-nitro-2-(1 H-indol-3-yl)ethane, indolylmethylthiohydroximate, indolylmethyldesulflo-GLS), and three indole GLS-derived phytoalexins: 4-methoxyindolecarbinol (4MI3C) (Fig. 8b), spirobrassinin (Fig. 8c), and raphanusamic acid (Fig. 8d). We identified three indole 3-acetonitrile-derived compounds previously reported in *Arabidopsis* and other Brassicaceae [41, 42]: Indole 3-carbaldehyde (I3CHO), Indole 3-carboxylic acid (I3CA) and Indole 3-carboxylic acid-hex (I3CA-hex) (Fig. 8e). Spirobrassinin was stimulated (about 10-fold) by CuCl_2_ in some *B. rapa* accessions, especially in the accession ‘Sampad’ exhibiting the highest levels following stimulation (see Table 1 for structural annotation). By contrast, Spirobrassinin was only weakly stimulated by CuCl_2_ in every *B. oleracea* accession (Fig. 8c, see also Supplementary Figure E).

In *B. oleracea*, the most striking metabolic consequence of CuCl_2_ stimulation was a 10-fold accumulation of IAA in most accessions (Fig. 8a). This was not observed in *B. rapa* accessions. Similarly, the phytoalexin raphanusamic acid was induced by CuCl_2_ at a much higher level in *B. oleracea* accessions than in *B. rapa* accessions (Fig. 8d). The highest accumulation of raphanusamic (20-fold increase) was observed in ‘po29-10’. Although not modulated by CuCl_2_ treatment, several other indole compounds displayed contrasted accumulation between accessions. Interestingly, the cabbage varieties ‘po29-10’ and ‘Vertus-Verius’ displayed not only high levels of indole GLS, but also high levels of their biosynthetic precursors (Fig. 8b).

### Aliphatic glucosinolate chemotypes and comparative analysis of *MAM* genes in *B. oleracea* and *B. rapa*

**Fig. 9:**
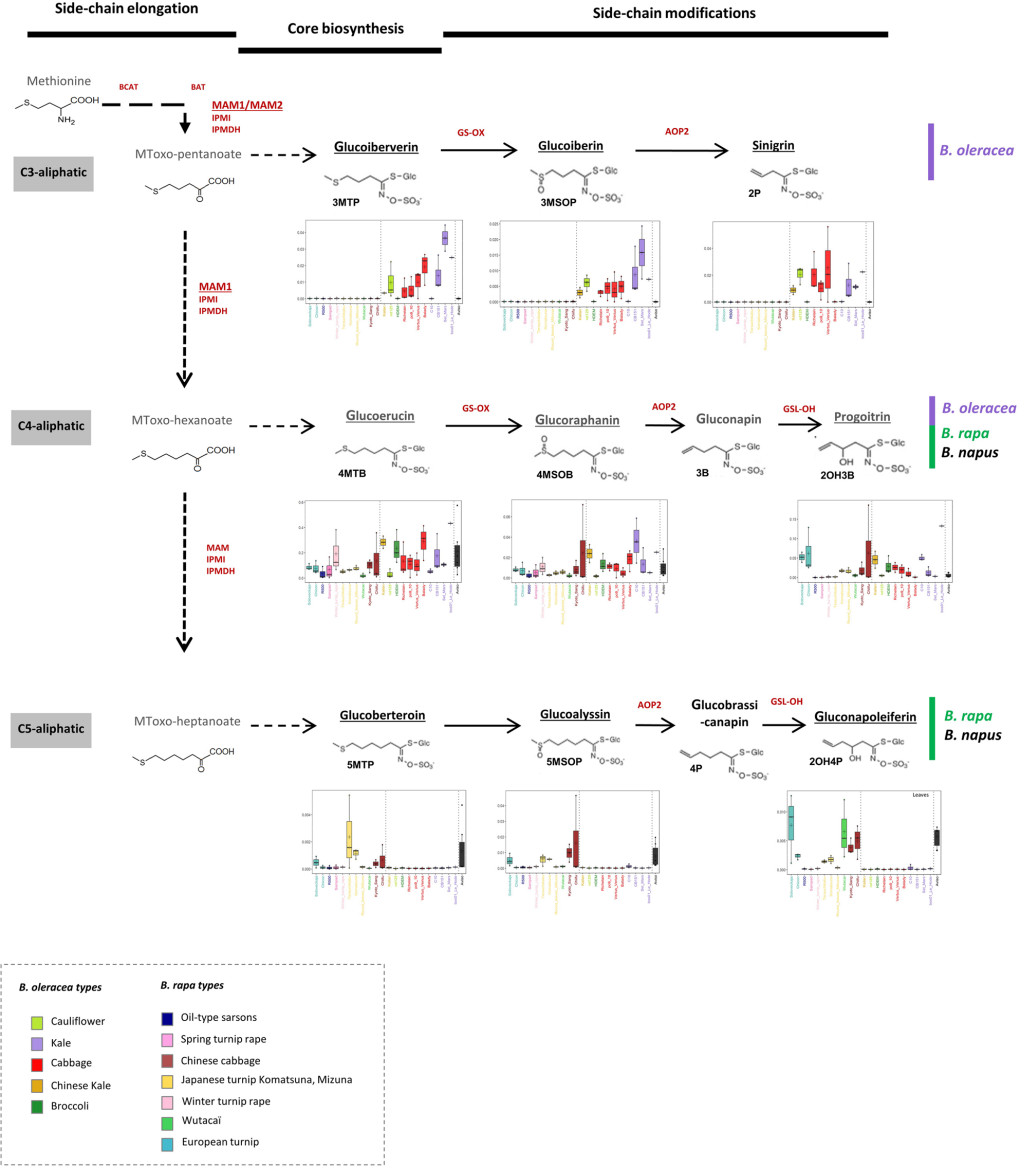
Distribution of 3C, 4C and 5C aliphatic glucosinolates in roots of *B. rapa* and *B. oleracea*. This figure shows a simplified schematic of the biosynthetic pathway of 3C, 4C and 5C aliphatic glucosinolates, highlighting genes involved (marked in red) and the distribution of these glucosinolates across different *B. rapa*, *B. oleracea*, and one *B. napus* accession. Dashed arrows indicate reaction intermediates not shown in the diagram. Boxplots illustrate the distribution of metabolite levels across accessions, with dashed lines separating species for clearer visualization. All details from the original schematic and distribution data are retained

Within the well-represented glucosinolate (GLS) families, 3C-aliphatic GLSs such as glucoiberin and sinigrin were exclusively detected in *B. oleracea* accessions, whereas 5C-aliphatic GLSs, including glucoberteroin and gluconapoleiferin, were restricted to *B. rapa* accessions (Supplementary Tables S5 and S6). A similar species-specific distribution is detailed for the root compartment in Fig. 9, where the *B. oleracea*-specific glucoiberverin (3C-aliphatic GLS) and the *B. rapa*-specific glucoalyssin (5C-aliphatic GLS) were identified (Fig. 9 and Supplementary Table S6). While 4C-aliphatic GLSs are commonly found in both species, statistical analyses revealed significant interspecies differences in their accumulation. Specifically, glucoraphanin levels were six-fold higher in *B. oleracea* leaves compared to *B. rapa*, whereas mean gluconapin concentrations were 20-fold higher in *B. rapa* leaves (Supplementary Table S5). Regarding indolic GLSs (e.g., glucobrassicin, hydroxyglucobrassicin, and desulfoglucobrassicin), *B. oleracea* accessions showed 2- to 3-fold higher contents on average compared to *B. rapa*. In contrast, the phenylalkyl GLS hydroxygluconasturtiin was more abundant in *B. rapa* accessions, with levels 6.8-fold higher than those in *B. oleracea* (Supplementary Table S5).

**Fig. 10:**
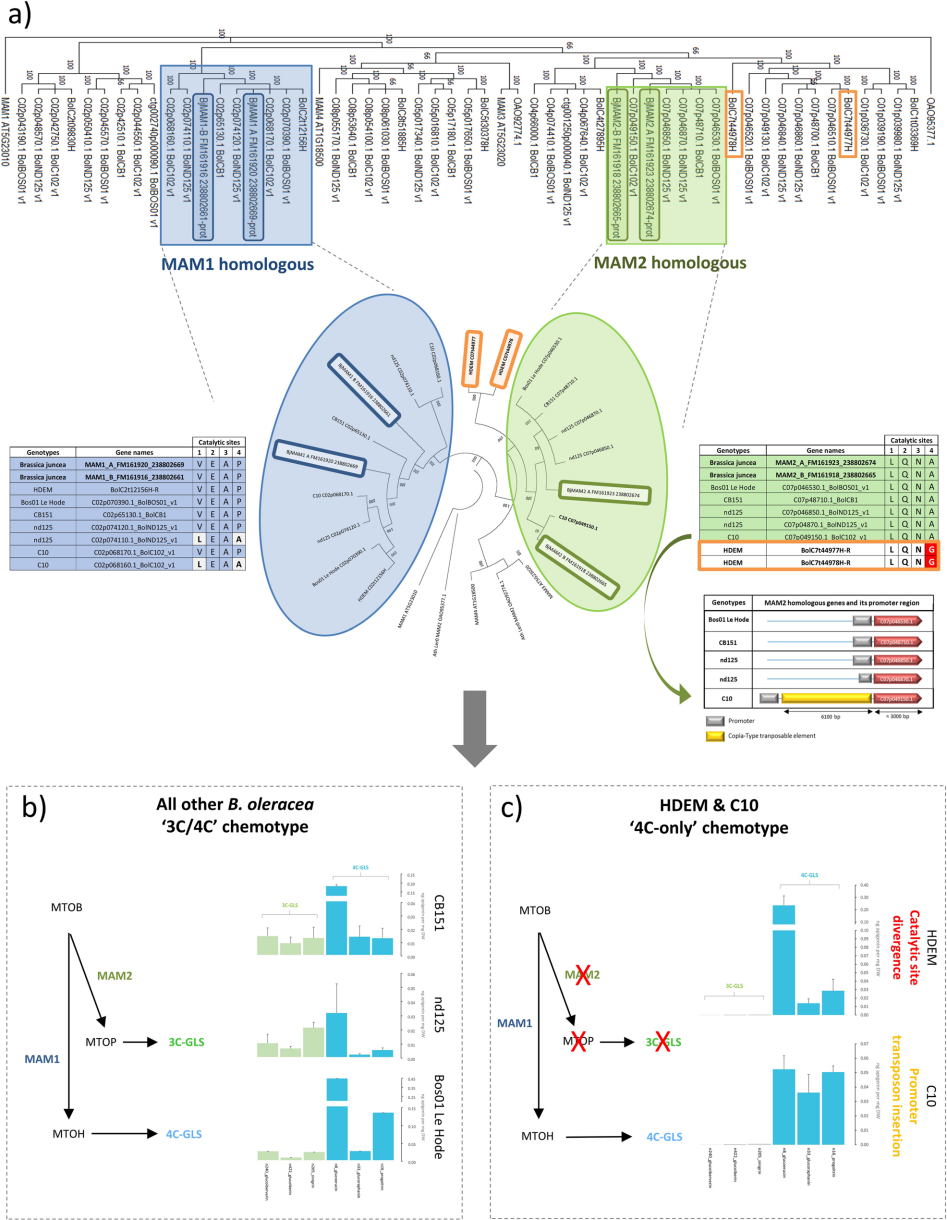
Genetic and functional basis of glucosinolate chain elongation diversity in *Brassica oleracea* accessions. (a) Composite panel summarizing molecular variation of *MAM* genes across *B. oleracea* accessions. A maximum parsimony phylogenetic tree was constructed using gene sequences obtained by BLAST of *B. juncea MAM1* and *MAM2* against multiple *B. oleracea* accessions, together with *MAM* sequences from *Arabidopsis thaliana* (Col-0 and Ler). The two *B. oleracea* gene clusters most closely related to *bjMAM1* and *bjMAM2* were extracted for a refined phylogeny. Both trees were rooted using *AtMAM1*. *MAM1* homologs are shown in blue (left) and *MAM2* homologs in green (right). Amino acid alignments of catalytic sites of *MAM2* homologs illustrate sequence divergence among accessions, including divergent residues in 'HDEM', and the insertion site of a Copia-type transposable element in the promoter region of the *MAM2* homolog in the 'C10' accession is indicated. Amino acid alignments of *MAM1* catalytic sites are also shown, highlighting conservation across accessions. (b) Canonical glucosinolate chain elongation pathway in accessions with a mixed 3C/4C chemotype ('CB151', 'nd125', 'Bos01 Le Hode'), where both *MAM1* and *MAM2* are functional. Schematic representation of the elongation cycle from MTOB to MTOH is shown together with histograms representing the accumulation levels of identified 3C- and 4C-aliphatic glucosinolates (mean ± standard error, ng apigenin equivalents per mg dried powder). (c) Glucosinolate chain elongation in 4C-only chemotypes ('HDEM' and 'C10'). These accessions lack a functional *MAM2*, as indicated by divergent catalytic residues in 'HDEM' and a Copia-type transposable element insertion in the promoter of the 'C10'-*MAM2* homolog. The schematic pathway is shown with histograms of 3C- and 4C-aliphatic glucosinolates (mean ± standard error, ng apigenin equivalents per mg dired powder); 3C glucosinolates are absent, consistent with the 4C-only chemotype. MAM1 catalyzes the entire elongation cycle from MTOB to MTOH, resulting in a distinct glucosinolate profile. MTOB : 4-methyl-thio-2-oxo-benzoate, MTOP : 5-methyl-thio-2-oxo-pentanoate, MTOH : 6-methyl-thio-2-oxo-hexanoate

Because 3C-aliphatic GLSs (glucoiberverin, glucoiberin, and sinigrin), which are specific to *B. oleracea*, were not detected in the accessions ‘HDEM’ and ‘C10’, we hypothesized that this phenotype might be associated with genetic variation affecting *MAM* genes, which are known to control side-chain elongation in aliphatic glucosinolates (Fig. 9). This hypothesis is consistent with previous studies in *Arabidopsis*, where allelic variation at *MAM* loci has been shown to determine glucosinolate chemotypes [48, 49]. In *Brassica juncea*, the allotetraploid genome contains two *MAM2* (*MAM2a* and *MAM2b*) and two *MAM1* (*MAM1a* and *MAM1b*) gene copies encoding enzymes involved in the biosynthesis of 3C- and 4C-aliphatic GLSs, respectively [36]. Based on this information, we searched for *MAM* homologs in the genomes of five *B. oleracea* accessions included in our core collection (‘nd125’, ‘Bos01 Le Hode’, ‘CB151’, ‘HDEM’, and ‘C10’) and reconstructed their phylogenetic relationships (Fig. 10a).

All *B. oleracea* accessions harbored at least one *MAM2* copy located on chromosome C07. Two accessions, ‘HDEM’ and ‘nd125’, also contained an additional recent tandem duplication of this gene. Notably, both *MAM2* copies in ‘HDEM’ (HDEM_C07t44977 and HDEM_C07t44978) displayed an alanine-to-glycine substitution at the fourth canonical catalytic residue (Fig. 10a), a change that may impair enzymatic activity and could explain the absence of 3C-aliphatic GLSs in this accession. In contrast, all accessions producing 3C-GLSs (‘Bos01 Le Hode’, ‘CB151’, and ‘nd125’) carried at least one *MAM2* copy with the four conserved catalytic residues. In the ‘C10’ accession, which exhibits a 4C-GLS chemotype, the single *MAM2* copy retained the canonical catalytic residues. However, analysis of its promoter region revealed a large 6,100 bp insertion that may affect gene expression (Fig. 10a). Approximately 5.6 kb of this insertion showed high similarity to a Copia-type retrotransposon, including both terminal LTRs and internal regions, suggesting a possible regulatory disruption.

All accessions also contained at least one copy of *MAM1* on chromosome C02 with a conserved catalytic site, consistent with the universal presence of 4C-aliphatic GLSs in these varieties (Fig. 10a). Two accessions, ‘nd125’ and ‘C10’, additionally harbored an ancient tandem duplication of *MAM1* displaying an imperfect catalytic residue pattern [36]. These degenerated copies likely lost their original MAM1 activity and may have undergone functional diversification.

Together, these results suggest that both coding-sequence variation and structural changes affecting *MAM2* gene regulation contribute to the observed differences in aliphatic GLS chemotypes among *B. oleracea* accessions. These relationships are summarized schematically in Fig. 10b and c.

## Discussion

In this study, we employed a metabolomic approach to comprehensively investigate the diversity of specialized metabolism in *B. oleracea* and *B. rapa*, the two parental species of oilseed rape, in combination with sequencing data to gain insights into the genetic basis of metabolic variation associated with glucosinolate metabolism. To expand the chemical catalogue of putatively identified specialized metabolites, CuCl₂ treatment was integrated into our experimental design to elicit the plant defense metabolome and significantly increase the number of ions differentiating the two *Brassica* species. Initial compound annotations were obtained by matching the dataset against an in-house phytochemical library and were subsequently expanded using GNPS-based molecular networking approaches. Annotation propagation within molecular subnetworks was performed manually, and all automated and inferred annotations were systematically validated by inspection of MS/MS fragmentation patterns, thereby limiting biases associated with chemoinformatic tools. This effort led to level 2 annotation, as defined by [33] and refined by [34] for 192 leaf metabolites out of 4,863 ions and 50 root metabolites out of 3,086, generating an unprecedented, high-resolution metabolomic dataset for these accessions that provides a valuable resource for the *Brassica* research community. This included various features belonging to glucosinolates and phenolic compound metabolic categories, as well as underexplored compound classes in *Brassica*, such as blumenols and phenolamides. The broadened chemical description presented here facilitates a more holistic understanding of phytochemical diversity, highlighting how it evolved since these two *Brassica* species diverged (about 4 million years ago), but also within cultigroups (Fig. 1). The observed variation in compound levels among accessions raises important questions regarding their potential biological functions but also the role of the evolutionary dynamics of the duplicated genes regulating their accumulation in root and leaf tissues. To illustrate this, we focus on selected classes of molecules specific to *B. rapa* or *B. oleracea*, whose distinctive accumulation patterns may provide valuable insights into species adaptation.

### Blumenol diversity in *Brassica*: ecological context and biosynthetic considerations

Blumenols are C₁₃-norisoprenoids belonging to the α-ionol-type megastigmane family, characterised by a skeleton derived from α-ionone and by variable oxidation patterns [50]. They are derived from carotenoids containing an ε-ring, such as α-carotene or lutein, through the action of carotenoid cleavage dioxygenases (CCD1 and CCD7), which contribute to the formation of C₁₃ apocarotenoid precursors of the ionone type, including blumenols A, B, and C [50, 51]. Blumenol A (vomifoliol) and blumenol B share the same hydroxylation pattern of the core structure, but differ in the saturation state of the side chain, which is fully saturated in blumenol B. Blumenol C also carries a saturated side chain similar to blumenol B, but lacks one hydroxyl group compared to blumenol B. To date, the specific enzymes responsible for these terminal transformations (hydroxylations, hydrogenation and formation of the saturated side chain, followed by aglycone modifications such as glycosylation and acylation) have not yet been formally identified or molecularly characterised.

Blumenols derivatives have been rarely reported in Brassicaceae, and this metabolic family remains poorly characterized in this lineage. To date, only one study on the wild cabbage species *Brassica fruticulosa* has reported the presence of five such compounds in leaves, including blumenol A [52]. In contrast, blumenol C derivatives, exhibiting diverse hydroxylation and glycosylation patterns, have been extensively described in the roots of dicotyledonous and monocotyledonous plants colonized by arbuscular mycorrhizal fungi (AMF) [51, 53]. Foliar accumulation of blumenol C has been proposed as a marker of AMF establishment, although the precise function of these compounds in the symbiotic process remains unclear [51].

Because Brassicaceae are considered a canonical non-mycorrhizal family, the detection of blumenol C derivatives in *B. oleracea* leaves in our study may seem surprising [54, 55]. However, this finding is not inconsistent with the presence, in the genomes of *Arabidopsis* and *Brassica* species, of genes encoding carotenoid cleavage dioxygenases CCD1 and CCD7, which are involved in the early steps of the norisoprenoid biosynthetic pathway. Moreover, Brassicaceae are known to interact with endophytic fungi, and AMF-like structures have been reported in *Arabidopsis thaliana*, *B. napus*, and *B. campestris* [56–58]. These ecological interactions suggest that blumenol C derivatives in leaves may reflect previously unrecognized biotic interactions rather than canonical AMF colonisation.

In our dataset, *B. oleracea* accessions accumulated both blumenol C hexoside and an acylated form, blumenol C malonyl hexoside. Malonylation of blumenols has been reported in other species, including *Medicago truncatula*, *Lotus japonicus*, *Allium porrum*, *Ziziphora clinopodioides*, and *Senecio jacobaea* [59–64], but the enzymatic mechanisms in Brassicaceae remain unidentified. Conversely, in our subset of *B. rapa* accessions, we observed the presence of specific blumenol A– and blumenol B–derived structures, including blumenol A hexoside, blumenol B malonyl hexoside, 6-hydroxyblumenol B malonyl hexoside, and demethylblumenol B hexoside. Unlike blumenol C, these compounds are not directly associated with AMF interactions. However, blumenol A has been shown to possess allelopathic activity in rice (*Oryza sativa* cv. Awaakamai), inhibiting shoot and root growth in cress [65], illustrating the diverse potential ecological roles of this metabolic class.

Taken together, the contrasting accumulation patterns of blumenols between *B. oleracea* and *B. rapa* indicate that metabolic divergence relies on differences in enzymatic capacities for the cyclohexenone core formation and downstream glycosylation or acylation, pointing to potentially species-specific biosynthetic pathways and biological functions. Thus, beyond the current metabolic characterisation, these observations open exciting avenues for investigating the genomic determinants of blumenol biosynthesis in *Brassica*. In our laboratory, we have initiated genetic program, including the use of introgression lines to dissect the genetic and enzymatic factors underlying the production of specific blumenol derivatives. This approach will allow us to link inter- and intraspecific metabolic variation to specific loci, gene families and potential regulatory networks controlling carotenoid cleavage, cyclohexenone formation, and downstream modifications.

### Phenolic compounds as markers of inter- and intraspecific diversity in brassicaceae

In the present dataset, isorhamnetin derivatives were detected exclusively in *B. rapa* accessions, consistent with previous reports in this species [66, 67] and with the observation that, when present in *B. oleracea*, these derivatives are typically found at very low concentrations in plant tissues [68].

By contrast, the identification of two kaempferol derivatives decorated with a rhamnoside (see Table 1 for structural annotation) in the leaves of the *B. oleracea* cabbage accession ‘CB151’ was unexpected. Although Brassicaceae species exhibit a wide diversity of flavonol glycosylation patterns, rhamnosides being common in *Arabidopsis* and the genus *Raphanus* to our knowledge, flavonol glycosylation in the genus *Brassica* has exclusively been associated with glucosides [69–71]. Genomic approaches could further elucidate the genetic determinants underlying this unique phytochemical variation. Specifically, these tools could help determine whether this trait arose from the introgression of genomic material from a genus related to *Brassica*, potentially during the breeding history of this variety.

The low or absent levels of hydroxycinnamoyl-malate esters in *B. oleracea* accessions observed in our dataset are consistent with findings from [72]. This pattern may suggest a deficit in the expression of enzymes responsible for hydroxycinnamoyl-malate transferase activity in this species. Moreover, the complete absence of these compounds in the two *B. rapa* accessions ‘R500’ and ‘Sampad’ suggests an allelic variation modulating malate transferase activity in these genotypes. This pattern mirrors the natural loss-of-function allelic variant reported for the *AtSNG1* gene, which encodes a sinapoylglucose: malate sinapoyltransferase (SMT) and is absent in certain *Arabidopsis* accessions such as Pna-10 [73]. *SNG1* belongs to the serine carboxypeptidase-like (SCPL) family, members of which often exhibit overlapping substrate specificities [74]. However, sequence analysis of genes homologous to *AtSNG1* in the genomes of ‘R500’ and ‘Sampad’ did not reveal any specific variations in the catalytic sites that could account for this loss of function. Consequently, further investigation into the presence or absence of functional *AtSNG1* homologs in these genomes would require experimental validation using purified recombinant enzymes to clarify their functional role.

### Diversity of CuCl_2_-triggered phytoalexin induction patterns

Spirobrassinin belongs to group B of indolic phytoalexins, as defined by [75], and is synthesized in *Brassica* species in response to certain phytopathogenic fungi. Spirobrassinin is derived from the indolic glucosinolate glucobrassicin via a biosynthetic pathway that is absent in *Arabidopsis*, involving an atypical PEN2-related myrosinase (BABG), a methyltransferase (DTCMT), and a cytochrome P450 enzyme (CYP71CR1) [28, 76]. Indolic phytoalexins from Brassicaceae exhibit variable antifungal activity, which depends on the capacity of different phytopathogenic fungal species to detoxify them [75].

Unbiased studies on the biosynthesis of this group of indolic phytoalexins can benefit from artificial stimulation of their production using oxidative treatments, such as CuCl₂ [24]. In this study, we show that CuCl₂-induced spirobrassinin synthesis varies significantly among *B. rapa* accessions and is undetectable in *B. oleracea* accessions. Given the potential role of spirobrassinin in fungal resistance, identifying the genetic determinants underlying this strong stress-dependent response in the *B. rapa* accession ‘Sampad’ and introgressing them into oilseed rape could be highly valuable.

Despite our efforts, we were unable to detect other phytoalexins reported in the literature as induced by abiotic or biotic stresses in *Brassica*, such as brassilexin and cyclobrassinin sulfoxide in *B. juncea* [77, 78], or isalexin, brassicanate, and rutalexin in rutabaga [79]. However, we observed a significant stress-induced stimulation of raphanusamic acid in *B. oleracea* genotypes. Raphanusamic acid, a cyclic thiocarbamate, is synthesised from the indolic glucosinolate 4-methoxyglucobrassicin *via* a pathway involving the atypical myrosinase PEN2 and glutathione-S-transferase GSTU13. Initially reported in *Arabidopsis* for its role in broad-spectrum fungal resistance [45], this compound has since been identified in several other Brassicaceae species [80].

In this study, raphanusamic acid increased 20- to 40-fold in response to CuCl₂ treatment across all *B. oleracea* accessions and an approximately 8-fold induction in the *B. rapa* accession ‘R500.’ Both raphanusamic acid and spirobrassinin are derived from indole glucosinolate-derived isothiocyanates, but their biosynthetic pathways diverge downstream. The contrasting CuCl₂-induced accumulation patterns of spirobrassinin and raphanusamic acid among *B. rapa* genotypes suggest that the regulation of these compounds occurs via distinct metabolic processes at the terminal enzymatic steps.

### Implications of *MAM2* gene and glucosinolate variation for chemotype diversity and ecological interactions

In *Arabidopsis*, where these allelic variations have been primarily documented, the GSL-ELONG cluster of *MAM* genes determines three chemotypes defined by the dominance of 3C, 4C, or 8C aliphatic glucosinolates [48, 49], and has been linked to resistance against specific herbivores [15, 16]. In *Brassica*, our study confirms a range of chemotypes across *B. oleracea* and *B. rapa* accessions, broadly consistent with previous reports [49].

The genetic basis of these chemotypes in *Brassica* is more complex due to lineage-specific duplication and functional diversification of *MAM* genes [49]. Our results suggest that the ‘4C-only’ chemotype in *B. oleracea* accessions ‘HDEM’ and ‘C10’ may arise from two independent genetic events: a coding-sequence mutation affecting the catalytic domain of *MAM2* in 'HDEM', and a large Copia-type retrotransposon insertion in the promoter of *MAM2* in 'C10' (Fig. 10). These findings highlight how both sequence variation and structural genomic changes can modulate GLS biosynthesis.

Transposable elements, which constitute roughly 40% of the *B. oleracea* genome, appear to play a major role in generating such genetic diversity [81]. Their insertion near or within genes can alter transcriptional regulation through novel cis-elements or epigenetic modifications [82–85], potentially influencing chemotypic variation in natural and cultivated populations.

These observations suggest that allelic and structural variation at *MAM2* loci may have functional consequences beyond GLS profiles, potentially affecting plant-insect interactions. Previous studies indicate that differences between 3C and 4C GLS chemotypes can alter isothiocyanate volatile emission, thereby influencing parasitoid activity on herbivores [86]. Future work, including heterologous expression of variant MAM enzymes and expanded genomic analyses, will be essential to clarify how *MAM2* variation shapes both metabolic phenotypes and ecological interactions in *Brassica*.

## Conclusion

The work presented here provides the first detailed and integrative overview of specialized metabolic diversity across a genetically structured panel of *B. oleracea* and *B. rapa* accessions. Beyond establishing a comprehensive metabolomic resource, this study illustrates how such a dataset can be exploited to explore the evolutionary, biochemical, and genetic bases of metabolic diversification within and between species.

Through an extensive annotation effort, including manual curation and molecular networking, we significantly expanded the range of compound classes considered in *Brassica*, notably incorporating underexplored families such as megastigmanes, phenolamides, and glucosinolate-derived indole phytoalexins. This refined chemical landscape constitutes a valuable reference for future phytochemical and functional studies. By combining metabolomic and genomic data, we were able to highlight striking species-specific and accession-specific metabolic signatures that likely reflect distinct evolutionary trajectories and adaptive strategies. The identification of blumenol glycosides in a non-mycorrhizal plant family, the contrasted patterns of flavonol glycosylation, the differential induction of phytoalexins upon CuCl₂ treatment, and the diversity of glucosinolate chain-length chemotypes all exemplify how this resource can reveal previously unrecognized biochemical capacities in *Brassica*. Importantly, these patterns do not merely describe variation, but point to concrete hypotheses regarding the enzymatic innovations, regulatory divergence, and ecological functions associated with specialized metabolites.

We further demonstrate that this panel enables the investigation of the genetic determinants underlying such metabolic contrasts, as illustrated by the analysis of *MAM* gene variation and its association with glucosinolate chemotypes. The availability of high-quality genome assemblies and resequencing data allows the exploration of both nucleotide-level polymorphisms and structural variations, including transposable element insertions, which appear to play a major role in shaping metabolic phenotypes in *Brassica*. In this sense, our dataset provides a framework for moving beyond descriptive metabolomics toward a mechanistic understanding of how metabolic diversity emerges and is maintained.

Finally, the development in our laboratory of genetic mapping populations derived from this panel will facilitate the functional validation of candidate genes and the *in planta* assessment of the ecological and agronomic relevance of specific metabolites. Altogether, this work establishes a foundation for integrative studies linking genotype, metabolism, and phenotype in *Brassica*, and illustrates how large-scale metabolomic resources can serve not only as descriptive catalogs but also as powerful tools for uncovering the evolutionary and functional logic of plant specialized metabolism.

## Supplementary Information

Supplementary Material 1. Table S1. Composition of the *Brassica rapa* and *Brassica oleracea* panel.

Supplementary Material 2. Table S2. Overview of leaves and root sample identification, treatments, and pooling of experimental blocks.

Supplementary Material 3. Table S3. Features detected in leaves of *B. rapa* and *B. oleracea* genotypes in ESI- and ESI+.

Supplementary Material 4. Table S4. Features detected in roots of *B. rapa* and *B. oleracea* genotypes in ESI- and ESI+.

Supplementary Material 5. Table S5. Structural annotation of all identified compounds in leaves and roots of *B. rapa* and *B. oleracea* genotypes.

Supplementary Material 6. Table S6. Statistical analysis of the specificity and inductibility of leaf features in *B. rapa* and *B. oleracea*.

Supplementary Material 7. Table S7. Statistical analysis of the specificity and inductibility of root features in *B. rapa* and *B. oleracea*.

Supplementary Material 8. Supplementary Figure A. Examples of base-peak UHPLC–HRMS chromatograms of *B. rapa* and *B. oleracea* leaf extracts.

Supplementary Material 9. Supplementary Figure B. ESI + MS/MS fragmentation of blumenol derivatives supporting structural annotation.

Supplementary Material 10. Supplementary Figure C. Hierarchical clustering of root metabolites with significant interspecific differences in *B. rapa* and *B. oleracea*.

Supplementary Material 11. Supplementary Figure D. ESI + MS/MS fragmentation of kaempferol rhamnoside derivatives illustrating structural assignment of glycosyl and acyl moieties.

Supplementary Material 12. Supplementary Figure E. Assessment of Intraspecific Diversity of Tryptophan-Derived Indolic Compounds in the Leaves of *B. rapa* and *B. oleracea* Accessions.

Supplementary Material 13. Supplementary Figure F. GNPS mirror plots supporting putative metabolite annotations.

## Data Availability

Sequencing data and associated materials referenced in this study are publicly available in the European Nucleotide Archive (ENA). Data corresponding to the 'C10', 'nd125', and 'Bos01_Le_Hode' accession are accessible under ENA project PRJEB91446 (https://www.ebi.ac.uk/ena/browser/view/PRJEB91446) while the genome of HDEM is available under assembly GCA_900416815.2 (https://www.ebi.ac.uk/ena/browser/view/GCA_900416815.2). All untargeted metabolomics raw data generated in this study (.mzXML files) have been deposited under the dataset identifier MSV000100402 in the MassIVE repository (https://massive.ucsd.edu/ProteoSAFe/static/massive.jsp) and are available with the FTP download link (ftp://massive-ftp.ucsd.edu/v11/MSV000100402/).
