# Supplementary figures and images for: Metabolomic analysis of a core collection of Brassica rapa and Brassica oleracea unveils unexpected chemical diversity with potential applications in chemical ecology and breeding
Source: BMC Plant Biol. 2026 Feb 17;26:569. doi: 10.1186/s12870-026-08269-4 (PMC13032581; doi:10.1186/s12870-026-08269-4)

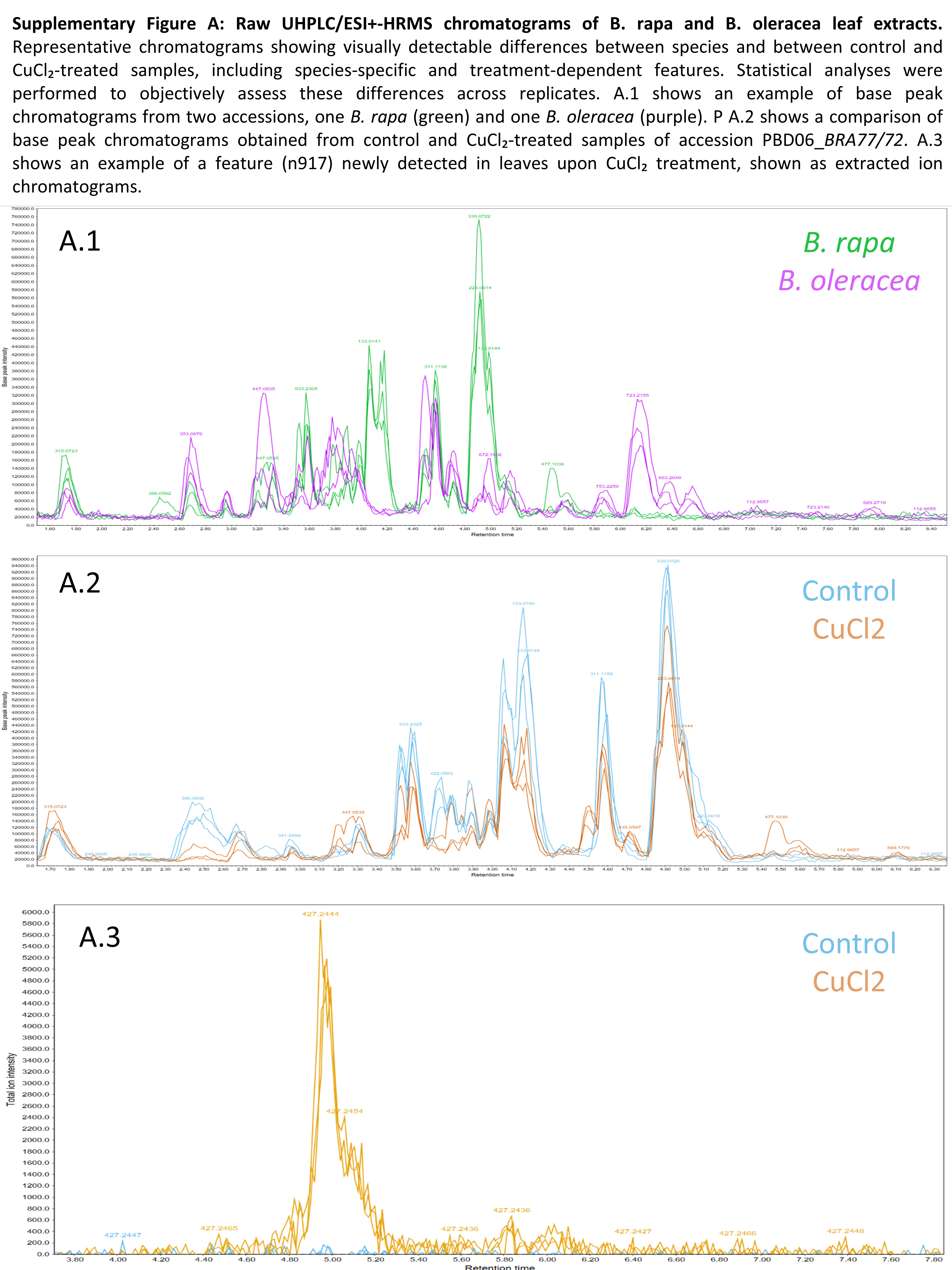

Supplement: Supplementary file 8 — Supplementary Material 8. Supplementary Figure A. Examples of base-peak UHPLC–HRMS chromatograms of B. rapa and B. oleracea leaf extracts. [file 12870_2026_8269_MOESM8_ESM.jpg]

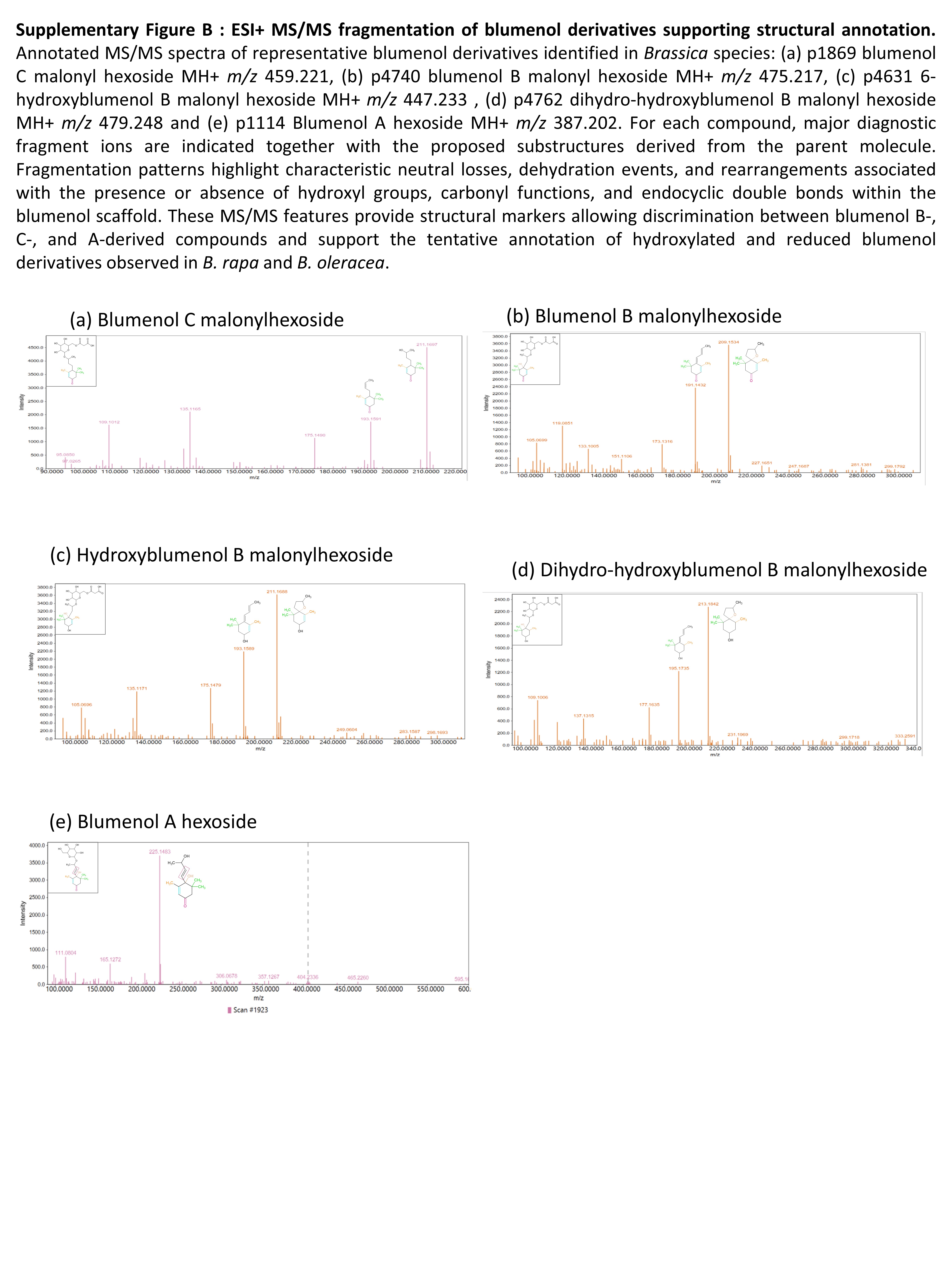

Supplement: Supplementary file 9 — Supplementary Material 9. Supplementary Figure B. ESI + MS/MS fragmentation of blumenol derivatives supporting structural annotation. [file 12870_2026_8269_MOESM9_ESM.jpg]

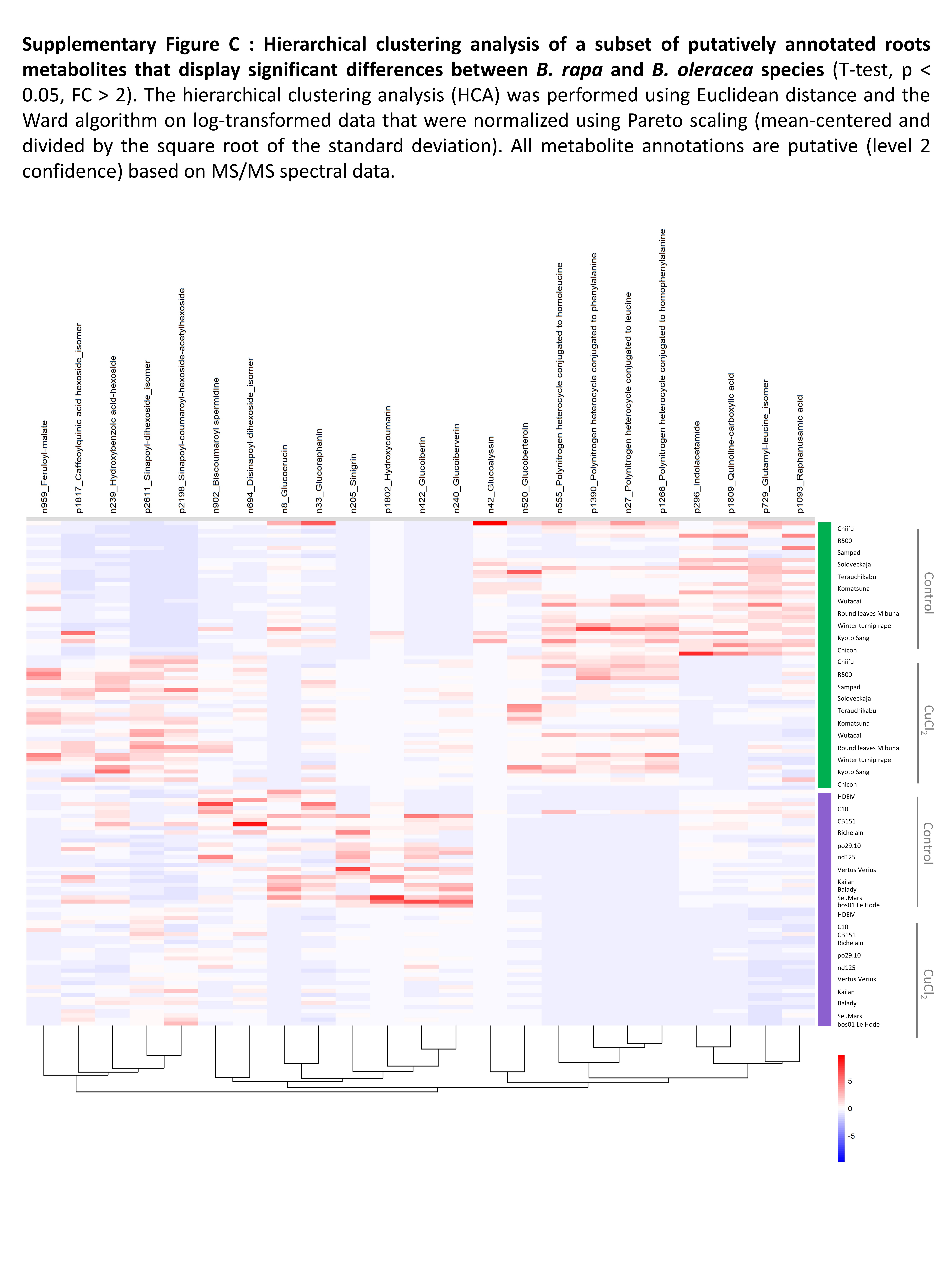

Supplement: Supplementary file 10 — Supplementary Material 10. Supplementary Figure C. Hierarchical clustering of root metabolites with significant interspecific differences in B. rapa and B. oleracea. [file 12870_2026_8269_MOESM10_ESM.jpg]

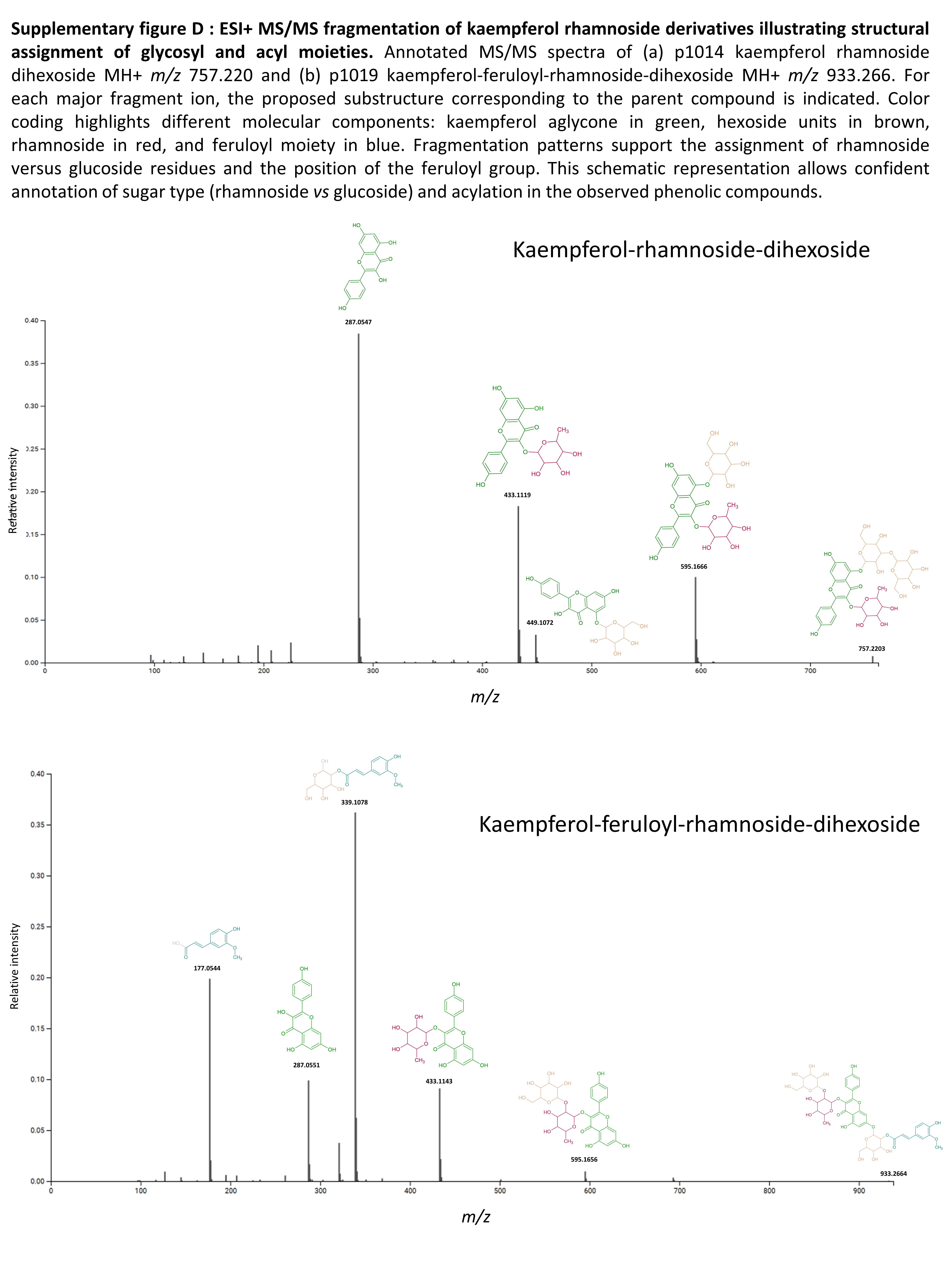

Supplement: Supplementary file 11 — Supplementary Material 11. Supplementary Figure D. ESI + MS/MS fragmentation of kaempferol rhamnoside derivatives illustrating structural assignment of glycosyl and acyl moieties. [file 12870_2026_8269_MOESM11_ESM.jpg]

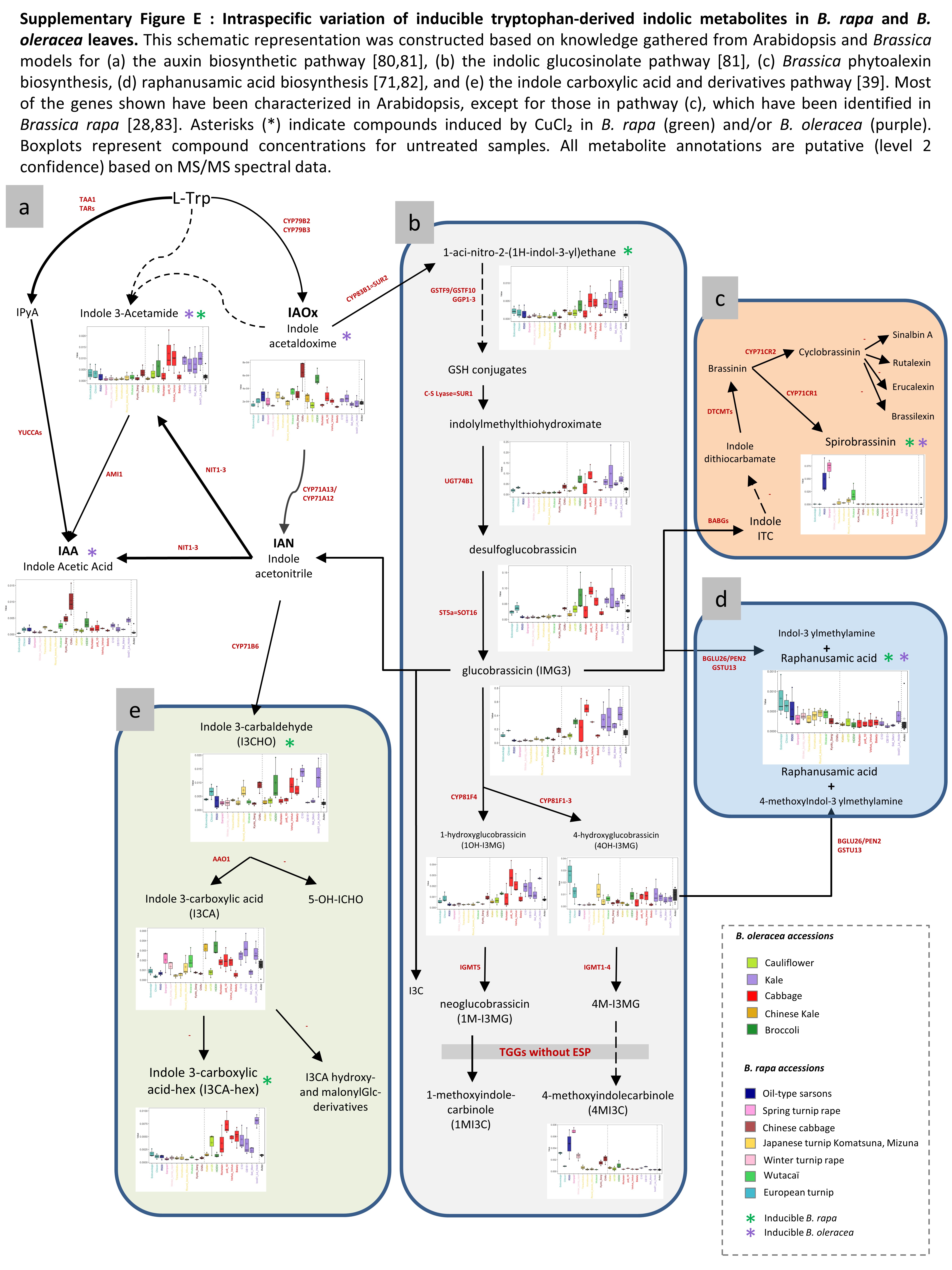

Supplement: Supplementary file 12 — Supplementary Material 12. Supplementary Figure E. Assessment of Intraspecific Diversity of Tryptophan-Derived Indolic Compounds in the Leaves of B. rapa and B. oleracea Accessions. [file 12870_2026_8269_MOESM12_ESM.jpg]
