# Supplementary material for: Metabolomic analysis of a core collection of Brassica rapa and Brassica oleracea unveils unexpected chemical diversity with potential applications in chemical ecology and breeding
Source: BMC Plant Biol. 2026 Feb 17;26:569. doi: 10.1186/s12870-026-08269-4 (PMC13032581; doi:10.1186/s12870-026-08269-4)

Supplementary Figure F : GNPS mirror plots supporting putative metabolite annotations. This figure compiles all MS/MS mirror plots obtained for features that matched reference compounds from the GNPS spectral library, using a cosine similarity score > 0.65 and at least three shared fragment ions. For each mirror plot, the experimental MS/MS spectrum acquired in this study is shown in blue, while the corresponding reference spectrum from the GNPS library is shown in red. These comparisons support the putative structural annotations assigned to the matched features. The complete list of fragment ions from the experimental spectra is provided in Supplementary Table S7, and the raw MS/MS data are available as .mzXML files deposited on MassIVE.

n54_Glucobrassicin

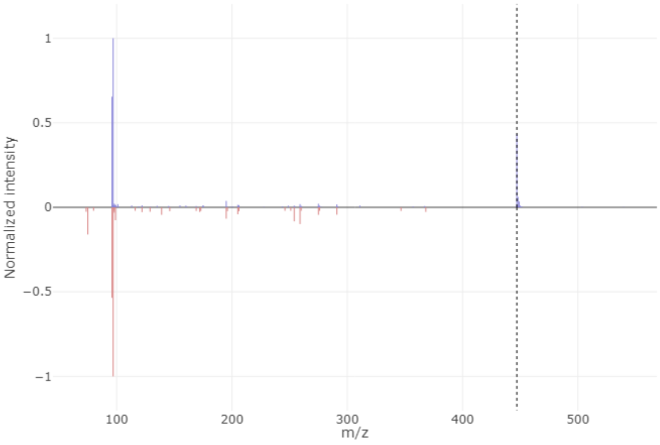

n60_Disinapoyl dihexoside

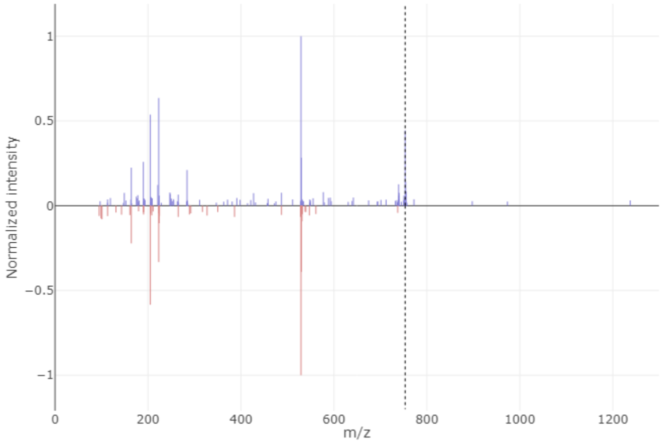

n244_Progoitrin

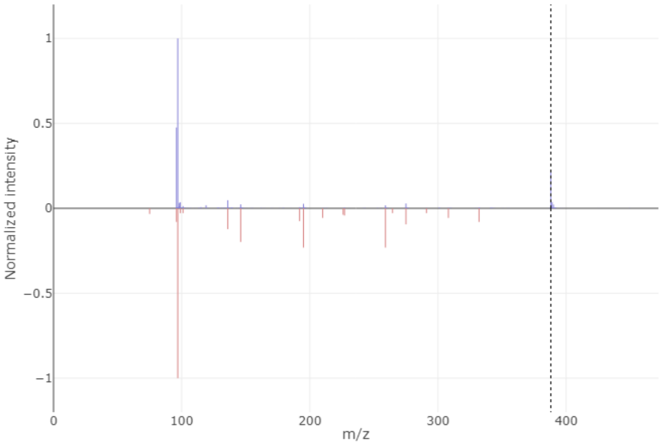

n385_Glucoraphanin

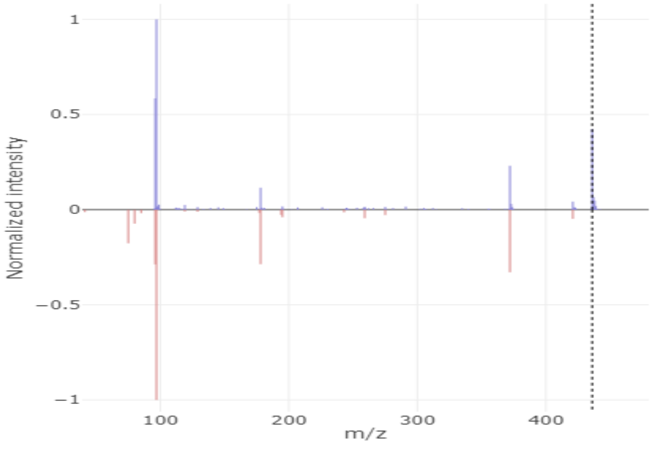

n426_Malyngic acid

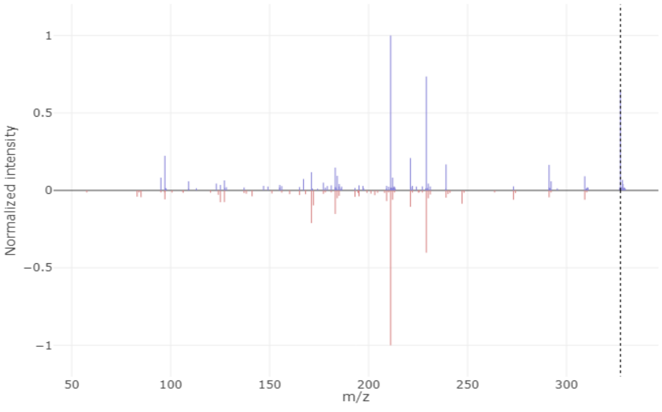

n435_Glucotropeolin

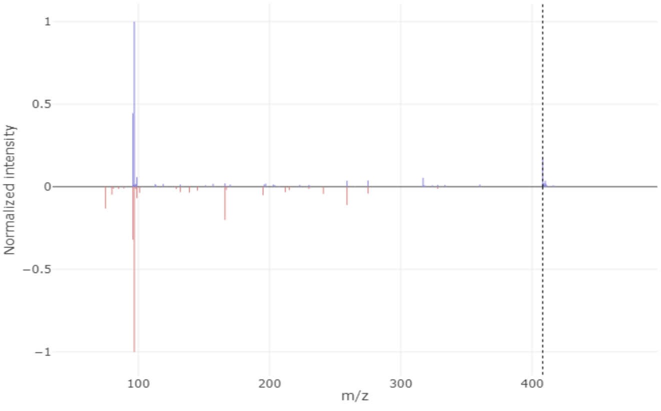

Supplementary Figure F (continued). GNPS mirror plots supporting putative metabolite annotations.

n542_Glucoiberin

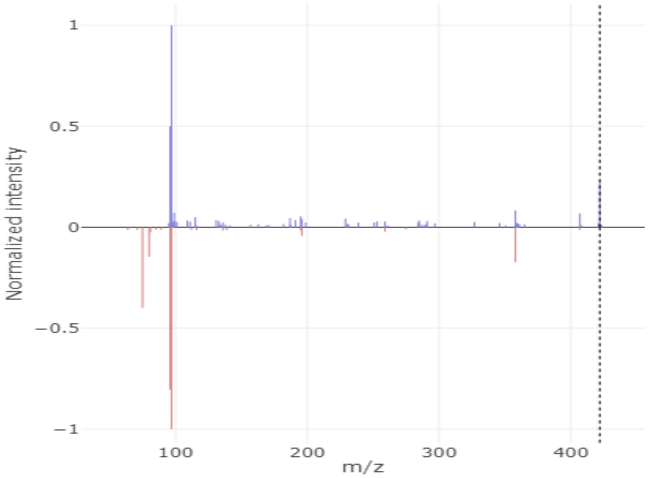

n546_Sinigrin

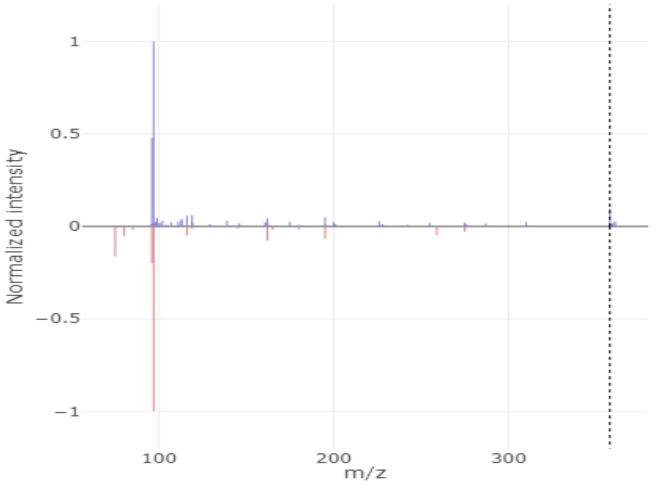

p50_Glycerol stearate

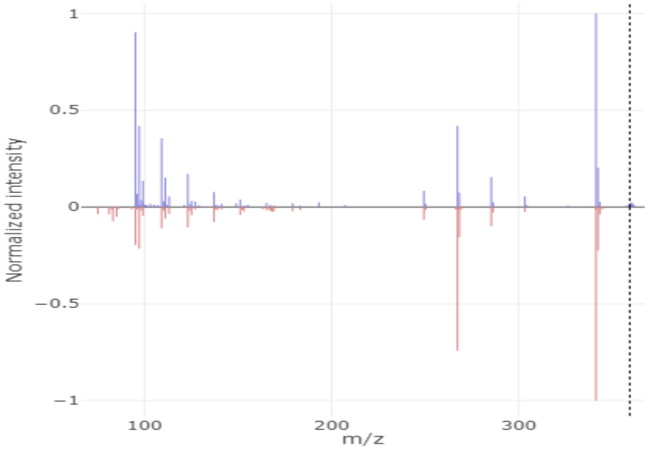

p132_Cocamidopropyl betaine

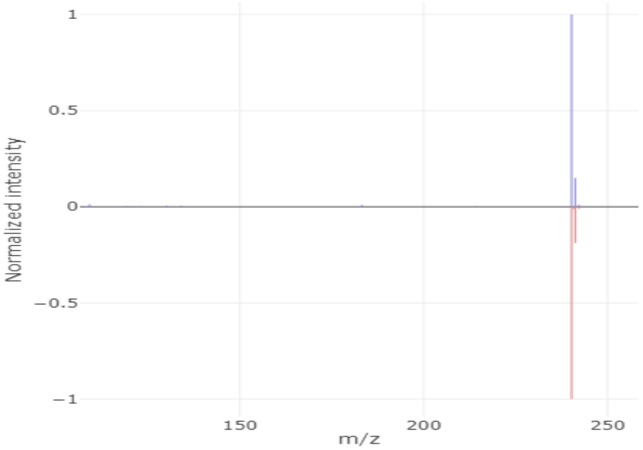

p270_Dibutyl adipate

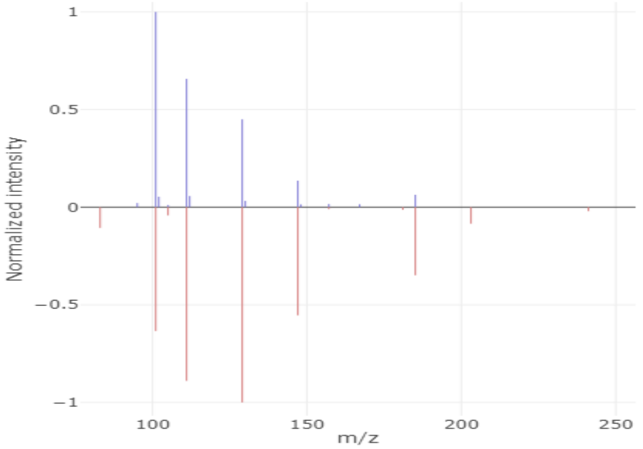

p402_Sinapoyl dihexoside

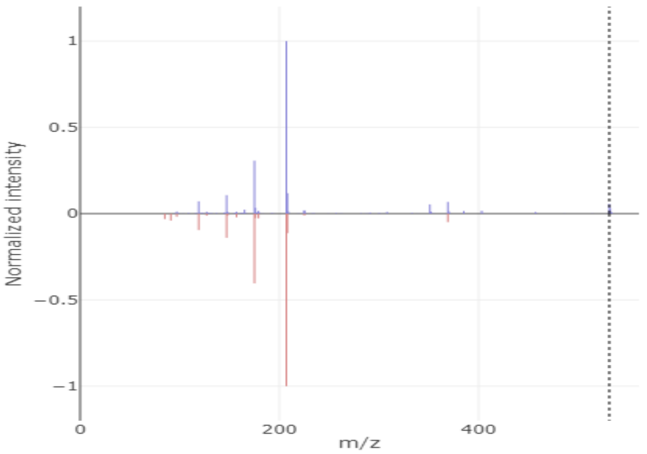

Supplementary Figure F (continued). GNPS mirror plots supporting putative metabolite annotations.

p544_Tyrosine

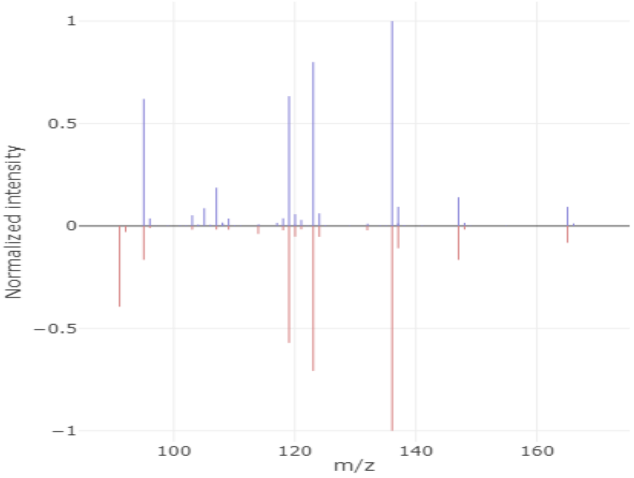

p565_Kaempferol hexoside

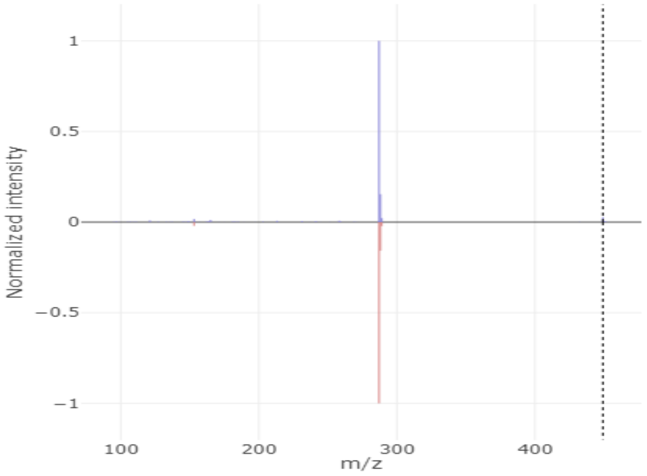

p607_Tryptophol

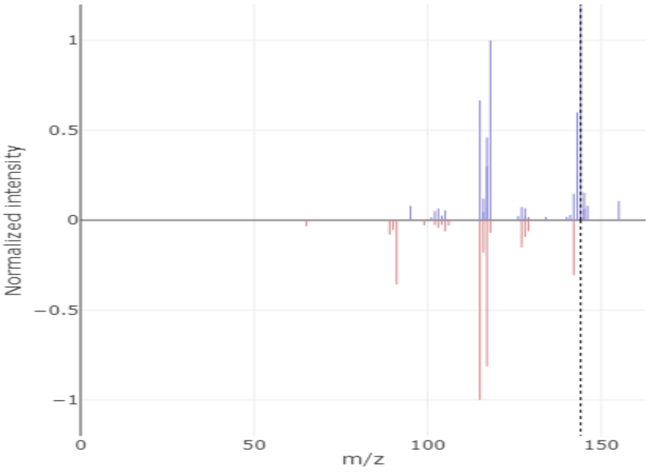

p617_N6-threonylcarbamoyladenosine

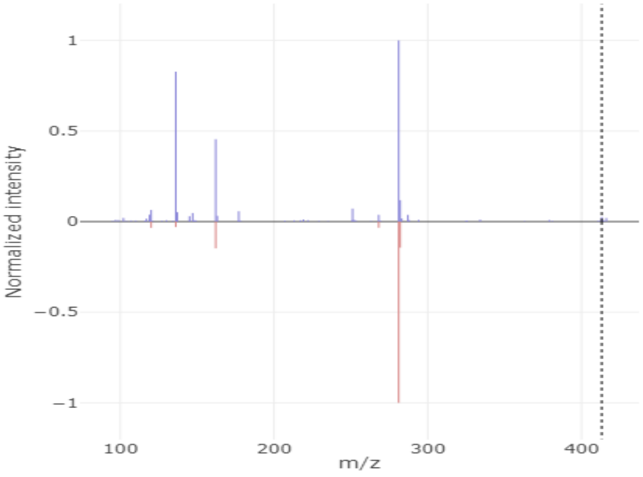

p623_Phenylalanylleucine

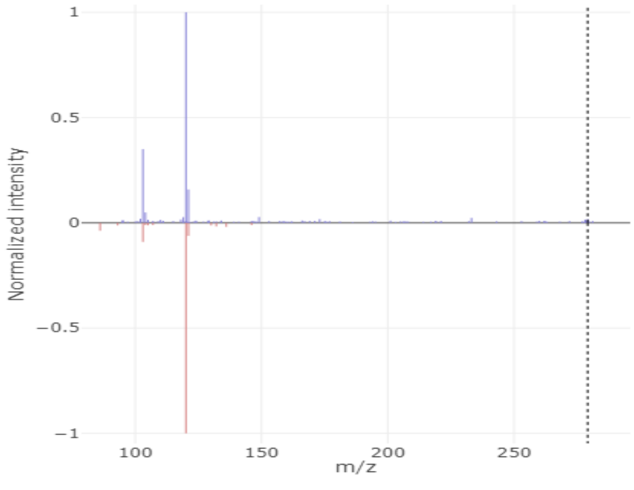

p720_Feruloyl-putrescine_iso

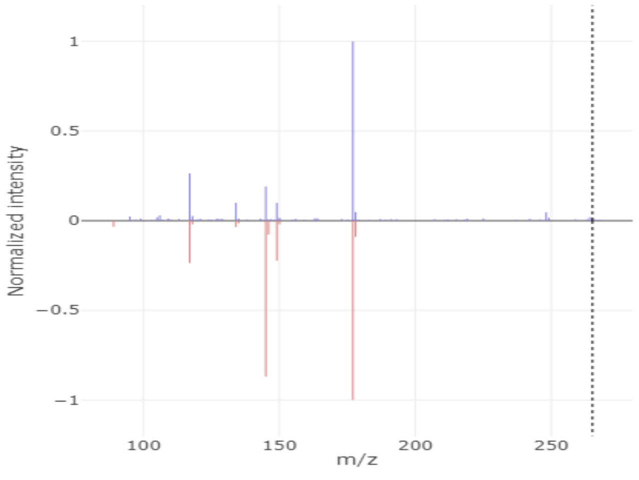

Supplementary Figure F (continued). GNPS mirror plots supporting putative metabolite annotations.

p758_Sinapoyl-dihexoside_iso

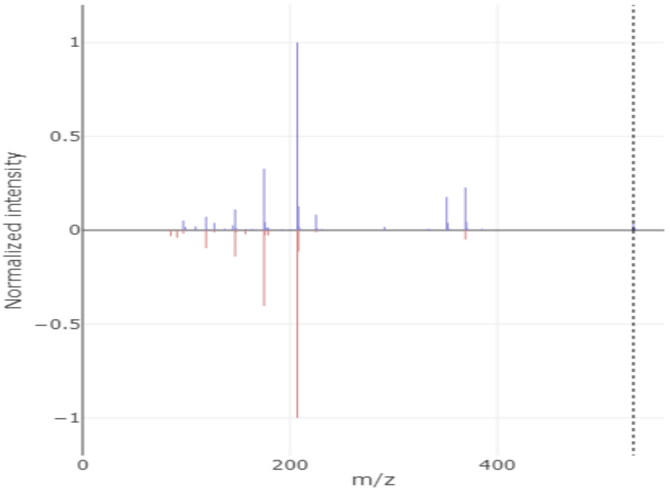

p859_Glycyl-Phenylalanine

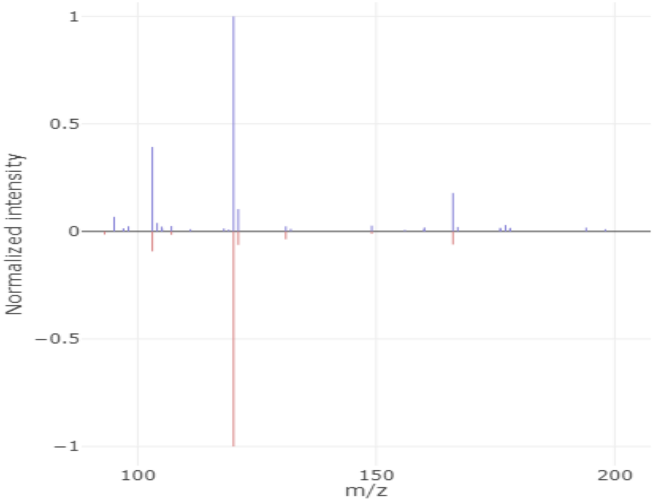

p872_Dihydroxybenzoic acid

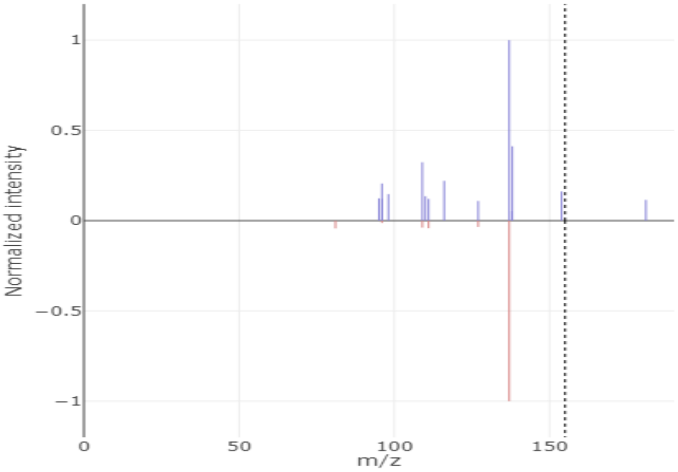

p932_Tyrosyl-Proline

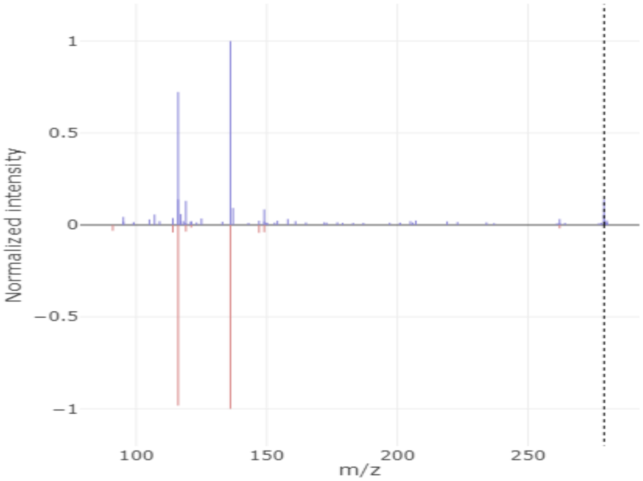

p1041_Indolacetamide

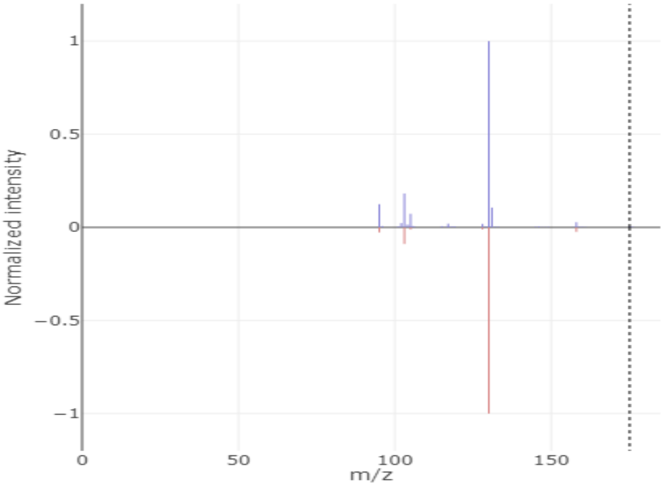

p1042_Kaempferol-rhamnoside-dihexoside

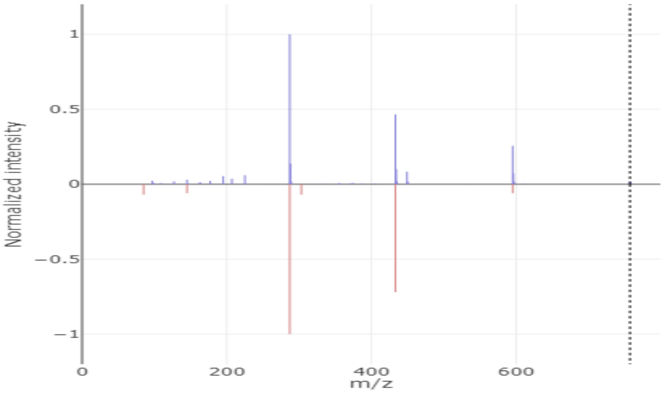

Supplementary Figure F (continued). GNPS mirror plots supporting putative metabolite annotations.

p1154_Syringaresinol hexoside

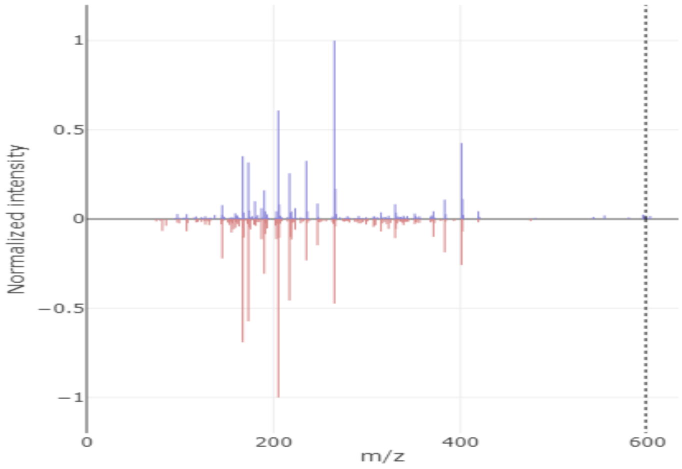

p1305_Isorhamnetin-dihexoside_iso

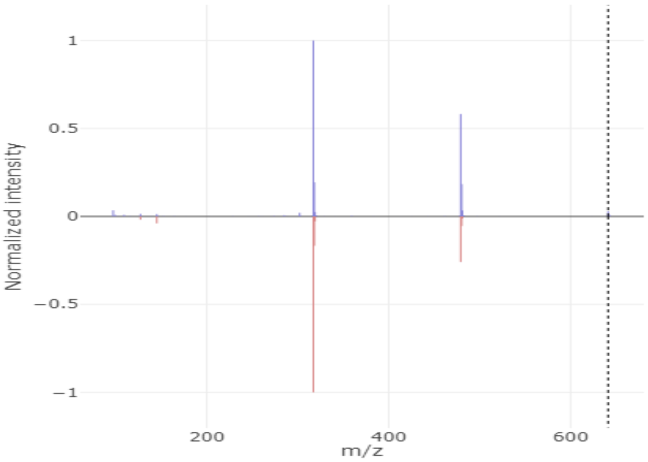

p1308_Feruloyl-putrescine_iso

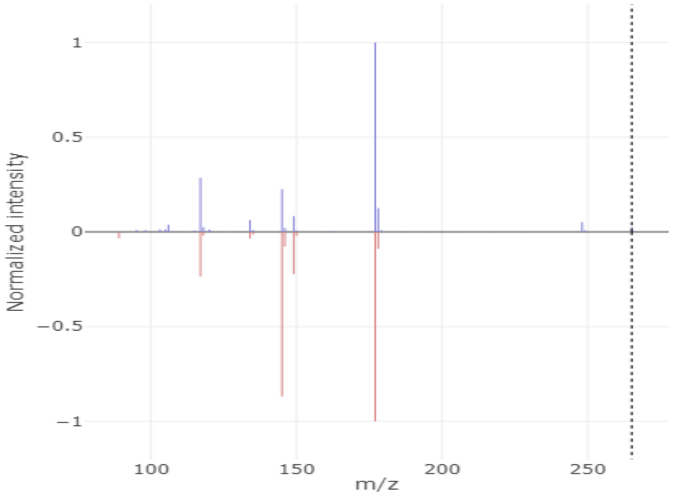

p1379_Quercetin-dihexoside_iso

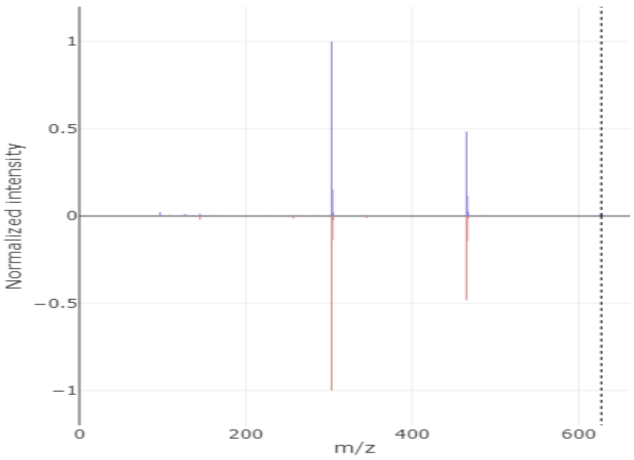

p1403_Coumaroyl-hexoside_iso

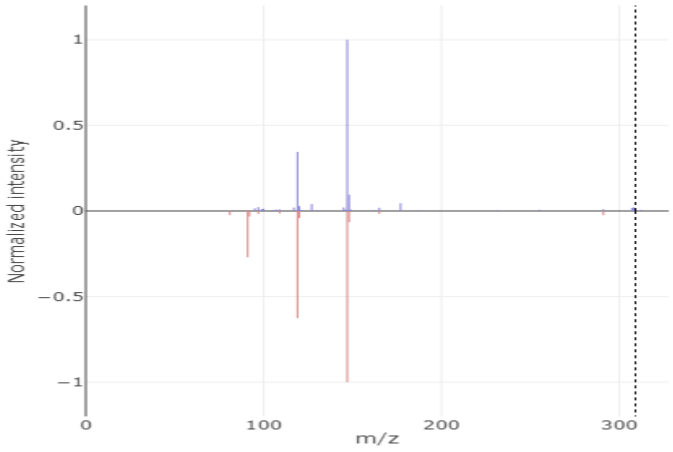

p1425_Dihydroxybenzoic acid

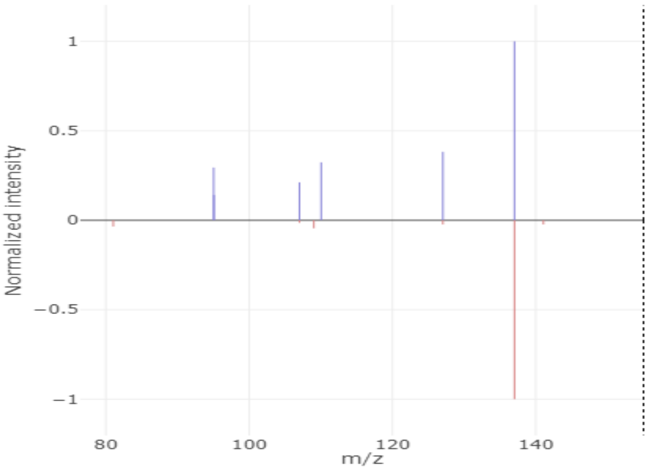

Supplementary Figure F (continued). GNPS mirror plots supporting putative metabolite annotations.

p1636_Succinoadenosine

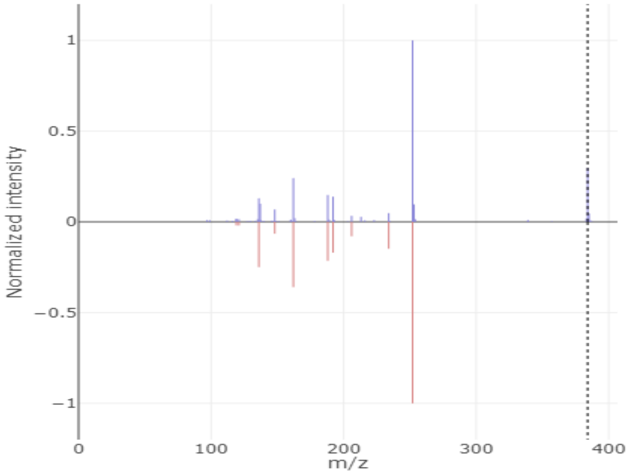

p1673_Kaempferol-dihexoside

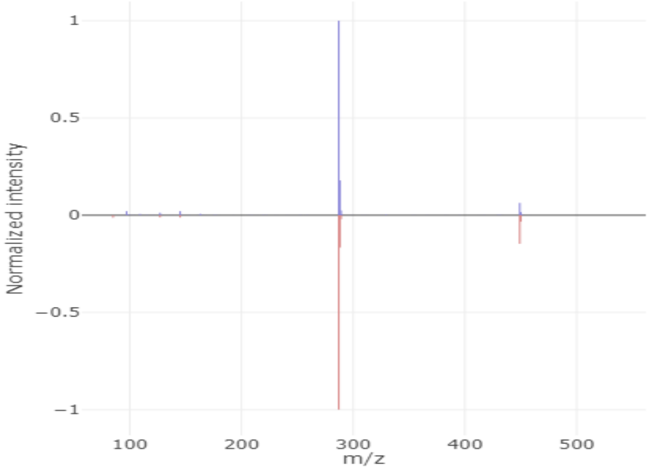

p1801_Jasmonic acid_isomer

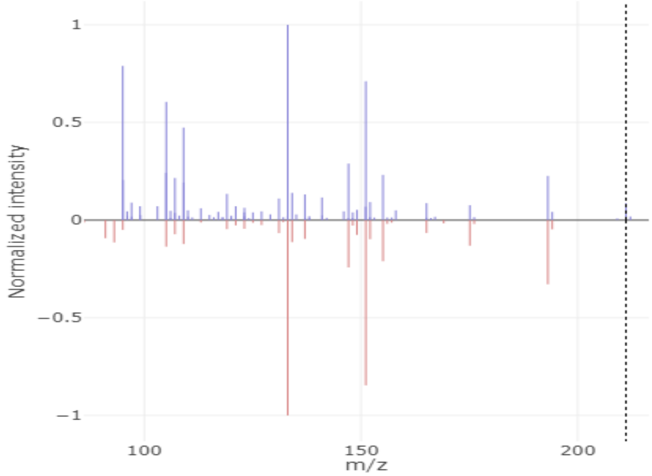

p2009_Indole carboxylic acid

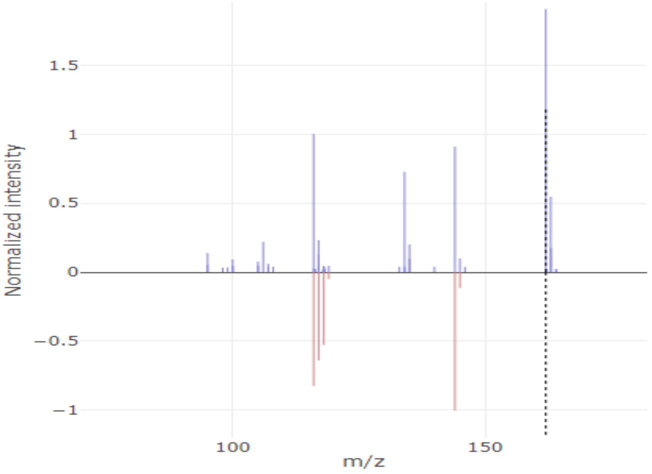

p2016_Coumaroyl-dihexoside

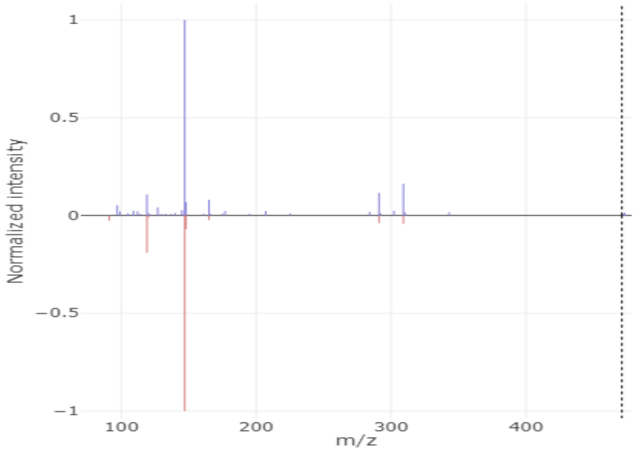

p2135_Isorhamnetin-dihexoside_iso

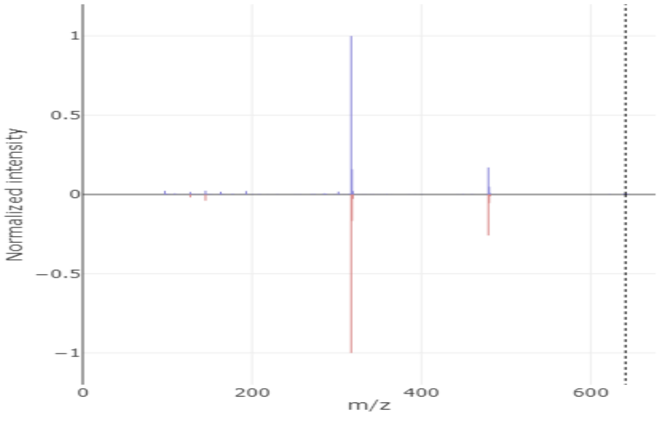

Supplementary Figure F (continued). GNPS mirror plots supporting putative metabolite annotations.

p2267_Caffeoylshikimic acid

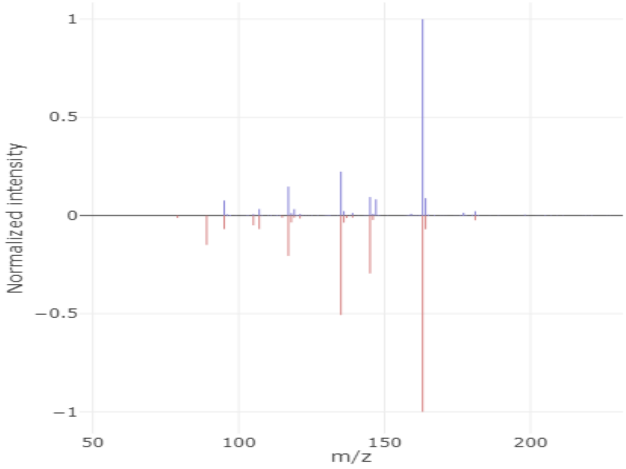

p2305_Hydroxybenzoic acid

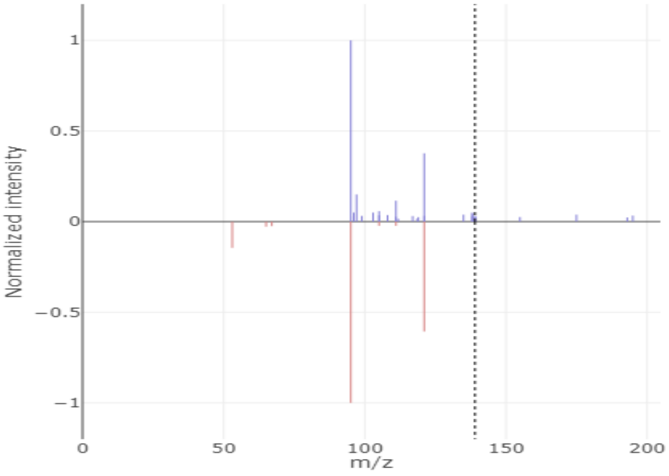

p2328_Spirobrassinin_iso

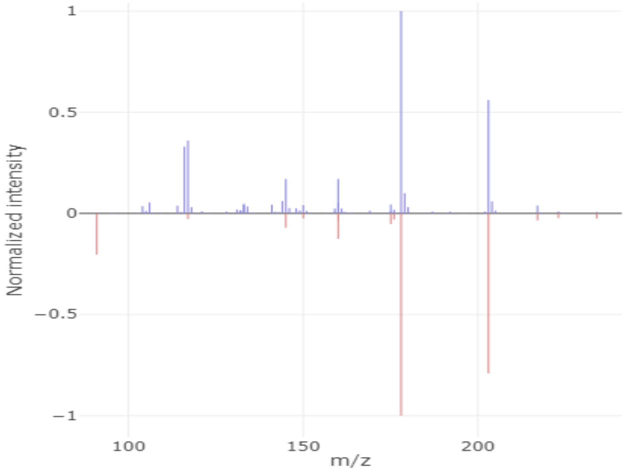

p2383_Jasmonoyl-isoleucine

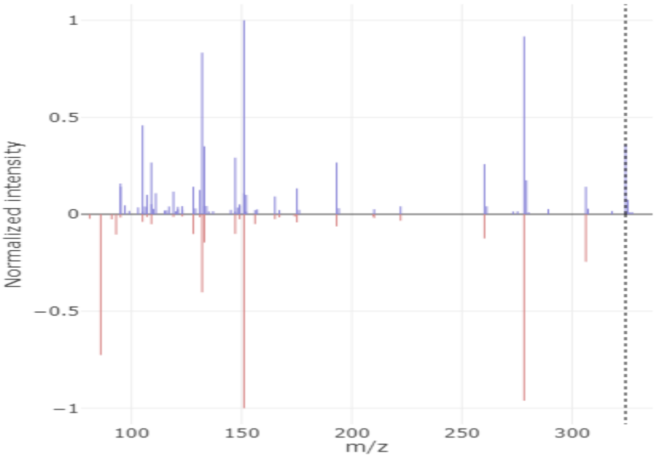

p2390_Glutamylphenylalanine

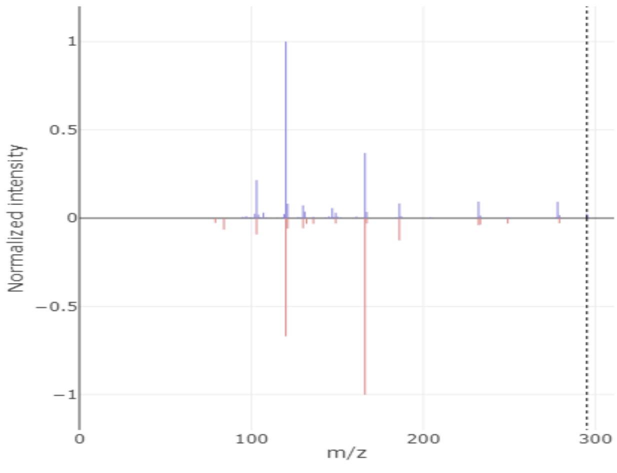

p2536_Spirobrassinin_iso

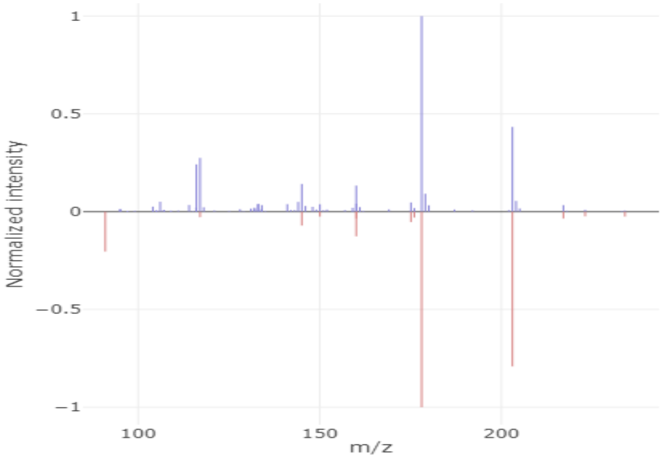

Supplementary Figure F (continued). GNPS mirror plots supporting putative metabolite annotations.

p2562_Methoxyindole-carbaldehyde

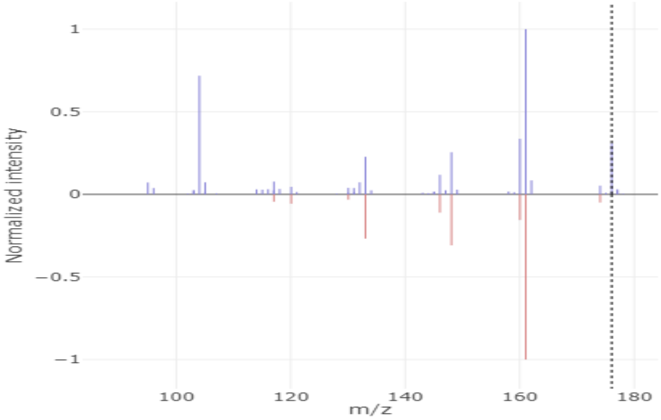

p2588_Quercetin-hexoside

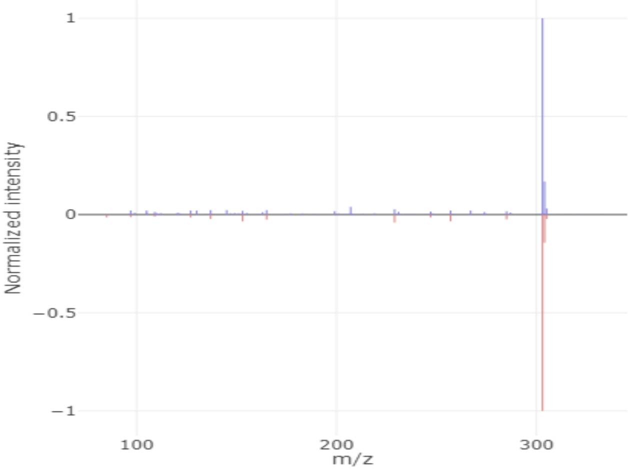

p2664_Sinapoyl-malate_iso

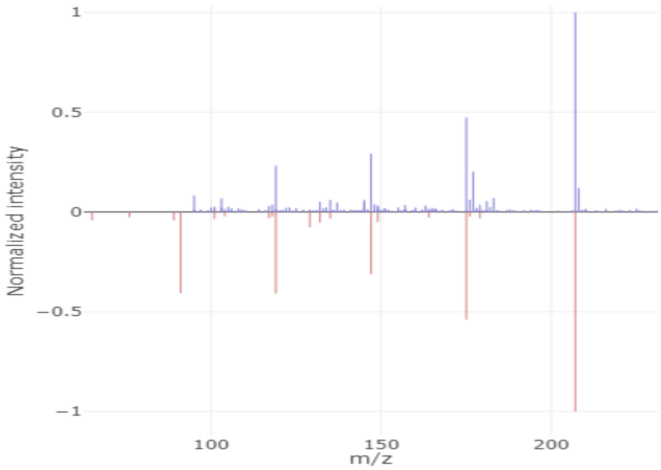

p3636_Spirobrassinin_iso

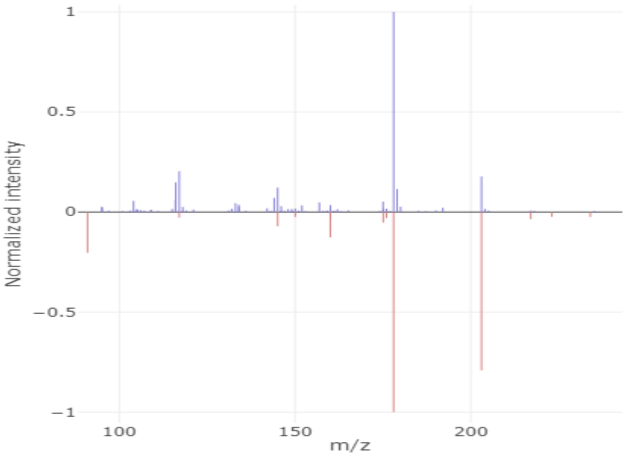

p3857_Jasmonic acid_iso

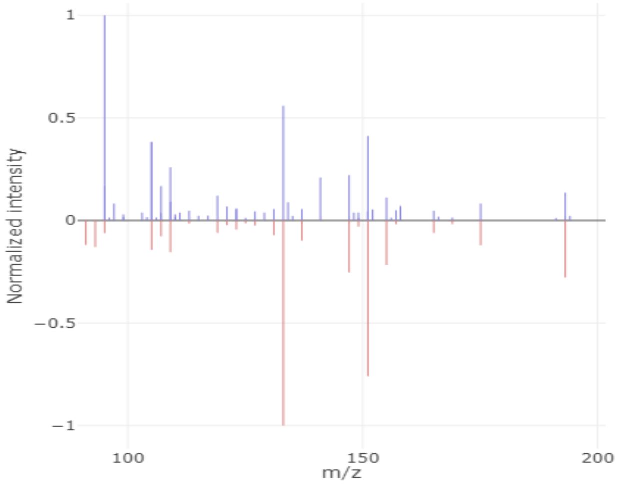

p3867_Sinapoyl-malate_iso

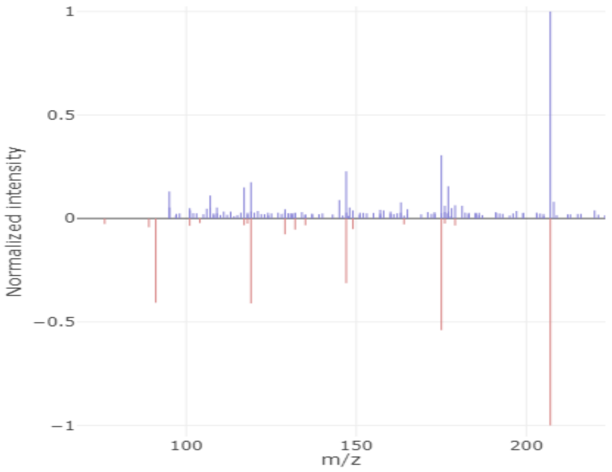

Supplementary Figure F (continued). GNPS mirror plots supporting putative metabolite annotations.

p3909_Riboflavin

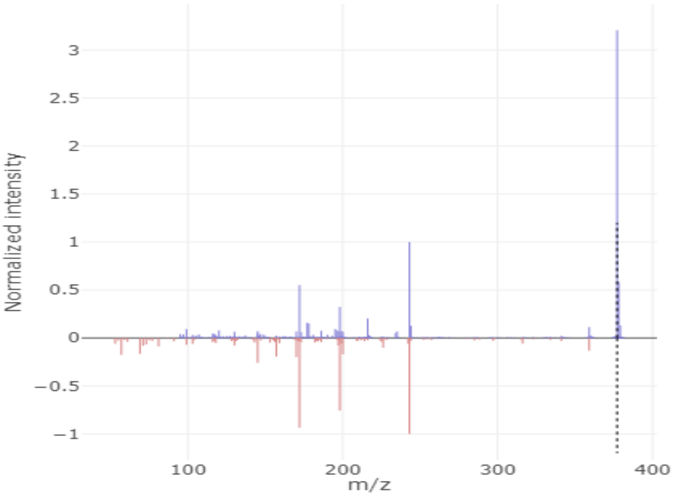

p3913_Formyl-kynurenine

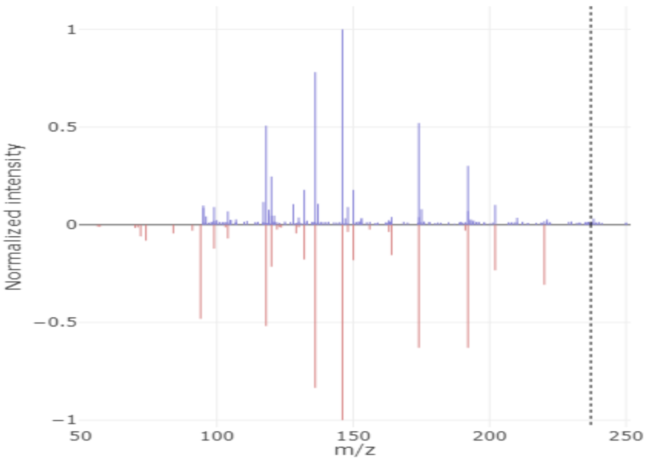

p4070_Coumaroyl-hexoside_iso

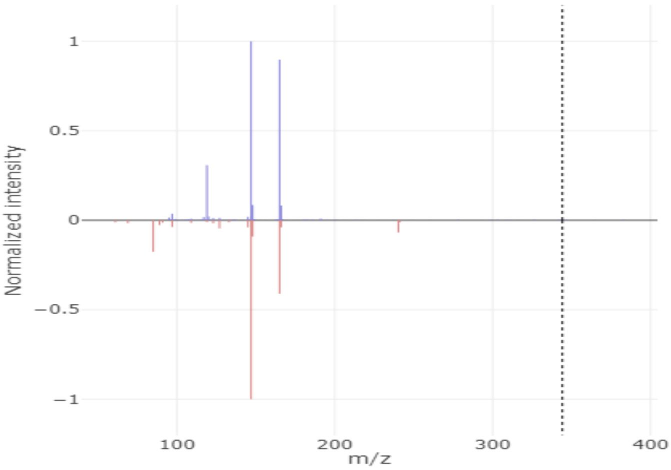

Supplement: Supplementary file 13 — Supplementary Material 13. Supplementary Figure F. GNPS mirror plots supporting putative metabolite annotations. [file 12870_2026_8269_MOESM13_ESM.pdf]
